# The epidemiology and risk factors for postnatal complications among postpartum women and newborns in southwestern Uganda: A prospective cohort study

**DOI:** 10.1371/journal.pgph.0003458

**Published:** 2024-08-07

**Authors:** Yashodani Pillay, Joseph Ngonzi, Vuong Nguyen, Beth A. Payne, Clare Komugisha, Annet Happy Twinomujuni, Marianne Vidler, Pascal M. Lavoie, Lisa M. Bebell, Astrid Christoffersen-Deb, Nathan Kenya-Mugisha, Niranjan Kissoon, J. Mark Ansermino, Matthew O. Wiens

**Affiliations:** 1 Department of Anesthesiology, Pharmacology & Therapeutics, University of British Columbia, Vancouver, British Columbia, Canada; 2 Institute for Global Health, BC Children’s Hospital Research Institute, Vancouver, British Columbia, Canada; 3 Department of Obstetrics and Gynaecology, Mbarara University of Science and Technology and Mbarara Regional Referral Hospital, Mbarara, Uganda; 4 Digital Health, BC Children’s Hospital Research Institute, Vancouver, British Columbia, Canada; 5 WALIMU, Kololo, Kampala, Uganda; 6 Department of Obstetrics and Gynaecology, Faculty of Medicine, University of British Columbia, Vancouver, British Columbia, Canada; 7 Department of Pediatrics, University of British Columbia, Vancouver, British Columbia, Canada; 8 Division of Infectious Diseases, Department of Medicine, Massachusetts General Hospital and Harvard Medical School, Boston, Massachusetts, United States of America; PLOS: Public Library of Science, UNITED STATES OF AMERICA

## Abstract

Sub-Saharan Africa accounts for two-thirds of the global burden of maternal and newborn deaths. Adverse outcomes among postpartum women and newborns occurring in the first six weeks of life are often related, though data co-examining patients are limited. This study is an exploratory analysis describing the epidemiology of postnatal complications among postpartum women and newborns following facility birth and discharge in Mbarara, Uganda. This single-site prospective cohort observational study enrolled postpartum women following facility-based delivery. To capture health information about both the postpartum women and newborns, data was collected and categorized according to domains within the continuum of care including (1) social and demographic, (2) pregnancy history and antenatal care, (3) delivery, (4) maternal discharge, and (5) newborn discharge. The primary outcomes were readmission and mortality within the six-week postnatal period as defined by the WHO. Multivariable logistic regression was used to identify risk factors. Among 2930 discharged dyads, 2.8% and 9.0% of women and newborns received three or more postnatal visits respectively. Readmission and deaths occurred among 108(3.6%) and 25(0.8%) newborns and in 80(2.7%) and 0(0%) women, respectively. Readmissions were related to sepsis/infection in 70(88%) women and 68(63%) newborns. Adjusted analysis found that caesarean delivery (OR:2.91; 95%CI:1.5–6.04), longer travel time to the facility (OR:1.54; 95%CI:1.24–1.91) and higher maternal heart rate at discharge (OR:1.02; 95%CI:1.00–1.01) were significantly associated with maternal readmission. Discharge taken on all patients including maternal haemoglobin (per g/dL) (OR:0.90; 95%CI:0.82–0.99), maternal symptoms (OR:1.76; 95%CI:1.02–2.91), newborn temperature (OR:1.66; 95%CI:1.28–2.13) and newborn heart rate at (OR:1.94; 95%CI:1.19–3.09) were risk factors among newborns. Readmission and death following delivery and discharge from healthcare facilities is still a problem in settings with low rates of postnatal care visits for both women and newborns. Strategies to identify vulnerable dyads and provide better access to follow-up care, are urgently required.

## Background

**Fig 1:**
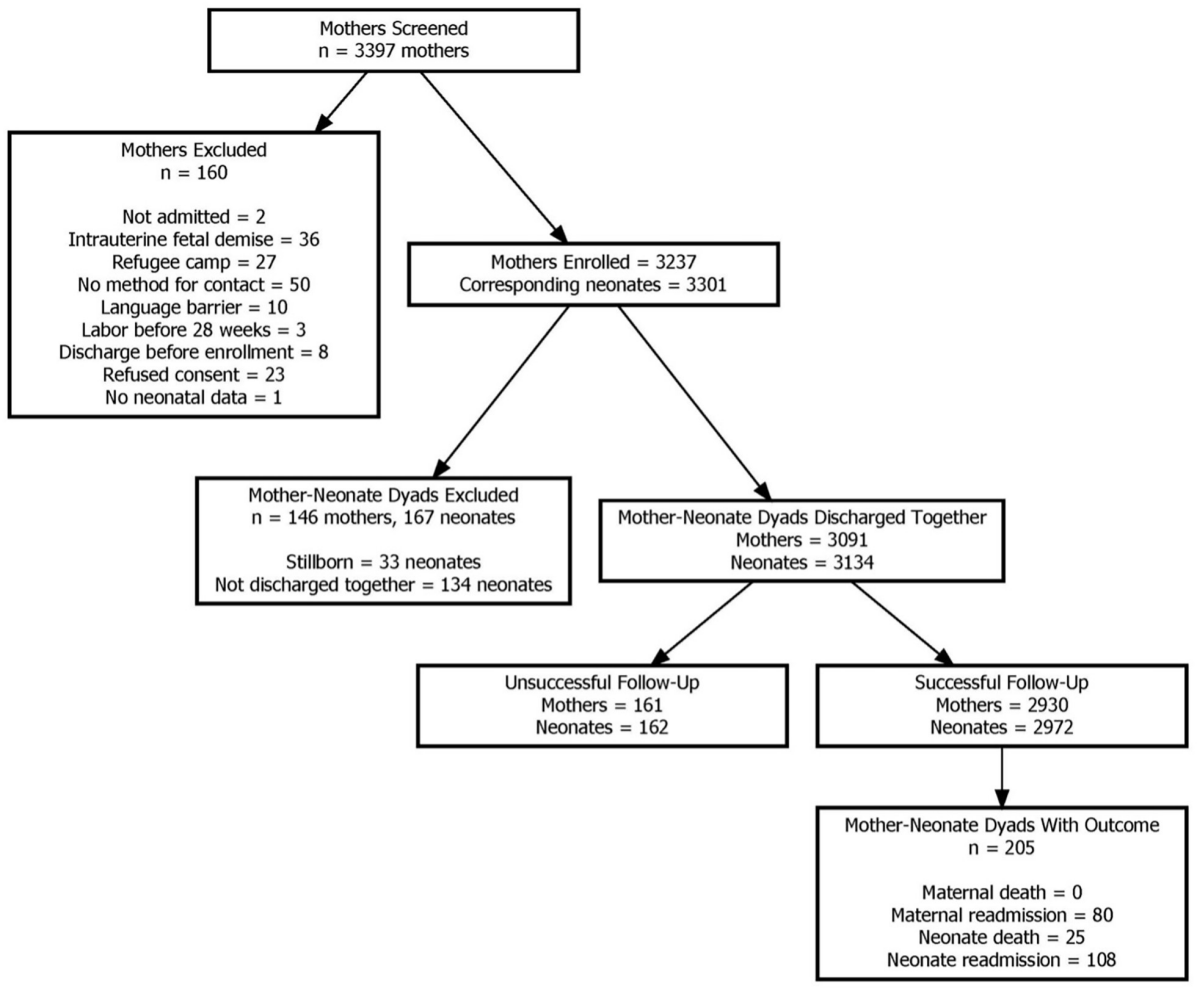
Flow chart of selection criteria for mother-baby dyads included in the study.

An estimated 290,000 postpartum women and 2.5 million newborns died from preventable causes worldwide in 2019, with Sub-Saharan Africa accounting for two-thirds of this figure [1–5]. The proposed United Nations Sustainable Development Goal target is to reduce the global maternal morality ratio to less than 70 per 100,000 births and the newborn mortality rate to 12 per 1000 live births by 2030 [6]. Despite significant progress in recent years, Ugandan estimates remain above this target with a maternal mortality ratio of 336 maternal deaths per 100,000 live births and newborn mortality rate of 19 deaths per 1000 live births [7]. The postnatal period accounts for nearly half of these deaths, which suggests a period of significant vulnerability for postpartum women and their newborns [7, 8]. Many of these deaths could be prevented by recognizing and treating complications earlier with quality postpartum care [9].

Maternal and newborn mortality estimates in southwestern Uganda are higher than the national average despite up to 70% of deliveries taking place in facilities [7, 10]. In this setting postpartum infection is a leading cause of mortality, and many of these infection-related deaths are preventable [11]. Although the causes of postpartum infection remain insufficiently studied, several studies have shown caesarean deliveries to represent a common and important risk factor [12]. Recent data from 39 countries in Sub-Saharan Africa estimate 5% of deliveries were completed via caesarean in 2018 with rates ranging from 1.4–50.7% between different countries in this region [13]. Furthermore, there is growing evidence that maternal and newborn outcomes are connected. Recent studies show maternal mortality and complications are associated with newborn mortality and failure to thrive and, early delivery to prevent maternal morbidity or morality can cause complications for the newborn due to prematurity [14–16]. Therefore, using information from both the mother and newborn offers a new approach to identify and address potentially preventable causes of postpartum complications [17]. Despite the magnitude and importance of this issue, postnatal complications among mothers and their infants in low- and middle-income countries (LMIC) like Uganda are poorly studied [18, 19].

Fragmented delivery and poor quality of care across the care continuum contribute to postnatal complications, but discharge processes are a neglected area of study [1, 12, 20–22]. The three delays model, well-established in obstetric literature, describes delays in the decision to seek care (type I), travel to the facility (type II) and in the provision of care at the facility (type III), helping to distinguish household, community and health system barriers to obstetric care in LMIC settings [23]. However, this model has shown limited functionality to improve transitions in between facility-based care and community-based care, including during the postnatal period [24–28]. The Ugandan Clinical guidelines, and World Health Organization guidelines, advise that women receive two or three postnatal care visits respectively, either at a facility or with a community health worker during the first six weeks following birth and discharge [29–31]. Even with these recommendations, postnatal care success is poorly defined in practice. Furthermore, contact rates among postpartum women and newborns vary greatly in most African settings, ranging between 8% and 85% [32, 33]. Therefore, improving postnatal care contact and quality could potentially prevent deaths during the postnatal period by recognizing and treating complications earlier [9].

Establishing evidence to inform recommendations surrounding the timing, frequency, and content of postnatal care for both mother and newborn, especially among the majority who receive routine discharges in low-resourced settings, is urgently needed. This exploratory analysis aimed to better describe the epidemiology of postnatal complications among postpartum women and newborns discharged together following a facility birth in Mbarara, Uganda. The primary objective of this study was to determine the incidence of, and risk factors for, maternal or newborn readmission or death within six weeks of discharge among postpartum women and newborns who were discharged together following facility delivery. A secondary aim of this study was to identify the most promising risk factors for readmission and death which could be used in future studies aimed at the development of a risk-prediction tool to identify those most in need of targeted post-discharge care interventions.

## Methods

### Study site and design

We conducted this prospective, observational study at the MRRH in Mbarara, Uganda. Mbarara, the largest city in the Southwestern region of Uganda has a population of approximately 195,000. MRRH is funded by the Ugandan Ministry of Health and is an approximately 300-bed academic hospital affiliated with Mbarara University of Science and Technology (MUST). It is the largest teaching and referral hospital for southwestern Uganda, with population of 4 million people living in a predominantly rural, agrarian setting. Approximately 9,500 deliveries were registered in the maternity ward in 2019.

### Enrollment and eligibility

We enrolled study participants between April 16, 2019 and March 31, 2020. Institutional review boards at the University of British Columbia (H18-02523), the Mbarara University of Science and Technology (14/09-18), and the Uganda National Council for Science and Technology (SS 4853) approved the study. All women and adolescent girls over 12 years of age admitted for delivery during this period were eligible for inclusion including twin and triplet gestations. Women presenting with intrauterine fetal demise at any gestational age or labor before 28 weeks based on last menstrual period (LMP) or best estimate were excluded. Women with language barriers (i.e., inability to speak or understand Runyankole or English), from a refugee camp, who could not be contacted by telephone for follow-up, or those discharged before enrollment were also excluded. Within the remaining sample, we were interested in postpartum women with liveborn infants who were discharged home together, directly from the maternity ward, following delivery. Those not discharged together (i.e., stillbirths and newborn or maternal intensive or high care unit admissions) were also excluded from analysis. These cohorts represent a different clinical risk category, and pathways of care and which will be examined in a separate study.

We used a consecutive sampling method during working hours, seven days a week. Participation was voluntary, written consent was obtained, and a copy of the signed consent form was provided to the study subject. Consent from mothers who were minors was obtained following local protocols, which was to consider them as emancipated minors with full independence to consent to participating in research.

### Procedures

This prospective cohort study aimed to enroll women presenting in labor at >28 weeks’ gestation who delivered liveborn infants and were routinely discharged together home with their infants. Following delivery, we obtained written consent to complete a structured questionnaire in-person and a follow-up questionnaire over the phone six weeks later. A total of 86 variables were collected and broadly categorized into five domains: (1) social and demographic, (2) pregnancy history and antenatal care, (3) delivery, (4) maternal discharge, and (5) neonatal discharge. Apart from discharge measurements, we prioritized gathering data from the hospital medical record, followed by interviews with the postpartum women and finally confirmation with the medical team if there were discrepancies, missing information, or questions the postpartum woman was unable to answer. With respect to discharge measurements, we obtained and recorded clinical data for both mother and their newborns on every dyad discharged together from the hospital. Blood pressure was measured using a Welch Allyn Vital Signs Monitor 300 Series (Welch Allyn, New York, USA). Oxygen saturation (SpO_2_) and heart rate was measured using the Masimo iSpO_2_ (Masimo Corporation, California, USA) and respiratory rates were measured using the RRate Application [34, 35]. Maternal hematocrit was quantified using a microhematocrit centrifuge. Random blood glucose was measured on mother and newborn using the FreeStyle Optimum Xceed (Abbott Healthcare, Massachusetts, USA). Anthropometric data of infants (length, weight, mid-upper arm circumference (MUAC), head circumference) were also measured and recorded. All dyads received routine care during admission and were discharged at the discretion of their medical teams.

Six weeks following discharge, women who were discharged with their newborns were contacted by phone to determine the status of the mother and newborn and timing and frequency of postnatal care visits. For children who died, the cause of death was collected, as reported by the caregiver (mother or other family member). In addition to vital status, details surrounding the timing, frequency and length of stay pertaining to readmissions and health seeking were also recorded.

Data were collected and managed using Research Electronic Data Capture (REDCap) tools hosted at the BC Children’s Hospital Research Institute in Vancouver, Canada [36].

### Objectives and outcomes

The sample size for this study was calculated based on an estimated incidence of composite maternal and neonatal death or readmission of 7%, taken from the study investigators previous work in Uganda [11, 37, 38]. The formula, [N = (p x 10) / I], was used to calculate the sample size where N = the required sample size, p = the number of predictor variables to be tested, and I = the incidence of the combined adverse outcome such that p > outcomes/10 to reliably assess individual risk factors in a multivariable model (11). Assuming a 10% loss to follow-up, we estimated a sample size minimum of 3200 women- and infant pairs were required (giving approximately 100 outcomes).

The primary outcome was death or readmission in either the mother or infant within six weeks of discharge. Secondary outcomes included the timing and frequency of routine postnatal care for both mother and newborn.

### Statistical analysis

Outcomes for mother and newborn were assessed independently. Risk factors were assessed using univariable and multivariable logistic regression within five prespecified domains of interest within the care continuum. Multivariable models included all variables within each domain and, where applicable, delivery mode (caesarean vs. vaginal) was included as an adjustment variable in the maternal discharge domain and the neonatal discharge domain. Multicollinearity was assessed using the variance inflation factor (VIF) in which variables were removed if the VIF exceeded 3 [39].

Kaplan-Meier survival curves were used to assess the time from discharge until maternal and neonatal death or readmission, and time from discharge until the first post-natal maternal and neonatal care visit.

All analyses were conducted using R Statistical Software (version 4.2.1). A p-value of <0.05 was considered statistically significant.

## Results

During the study period, 2930 dyads, or mother-infant pairs (2930 postpartum women and 2972 newborns) were enrolled, discharged together routinely and followed up. The characteristics of women who were enrolled but were not discharged with their infants (n = 146 postpartum women), and those who were lost to follow-up (n = 161 postpartum women) are reported in S1 Table. The characteristics of those lost to follow-up were largely similar to those discharged together. However, among those with a stillbirth or newborn admission, a greater proportion of postpartum women were diagnosed with medical complications such as pre-eclampsia or eclampsia, hypertension and antepartum hemorrhage. In addition, there were more caesarean deliveries and preterm deliveries observed in this group. The timing and causes associated with outcomes in this group are clinically distinct from those discharged together with their infants, and we plan to examine this further in future studies.

Of the 2930 postpartum women who had successful post-discharge follow-up, most were married (93.9%) and lived with the father of the infant (87.8%). The mean maternal age was 25.7 years (SD 5.6), median (IQR) parity was 2 (1–3), and 309 (10.5%) were HIV-infected, with one-third of these being diagnosed during pregnancy (Table 1). Among eligible participants, 1168 (39.9%) women gave birth by caesarean, and 149 (5.1%) were reported to be preterm (<37 weeks). Of the 2972 newborns discharged with their mother, 2880 were singletons, 98 twins and 6 sets of triplets. Five and two infants who were part of twin and triplet births respectively were stillborn, and three infants who were part of the twin births were admitted to neonatal intensive care. Overall, 232 (7.8%) of the discharged newborns weighed less than 2500g at birth and 496 (16.7%) required resuscitation following birth, of whom 34 (7.4%) received oxygen during their resuscitation. Among dyads discharged together, the median time between delivery and discharge was 18 (11–23) hours for those delivered vaginally and 67 (59–79) hours for those delivered by caesarean.

**Table 1:** Demographics of all eligible dyads discharged together with follow-up (n = 2930 mothers and 2972 newborns) and among these, dyads who had either a maternal or neonatal outcome at follow up (n = 80 mothers and 133 newborns).

Characteristics	All Mother/Newborn Dyads	Dyads with Maternal or Newborn Outcome	Total N Missing (% All Dyads)
**Maternal Demographics**			
Maternal age (years), mean (SD)	25.7 (5.6)	26.6 (5.8)	4 (0.14%)
Parity, median (Q1, Q3)	2 (1, 3)	2 (1, 4)	0 (0%)
Married, n (%)	2751 (93.9%)	75 (93.8%)	0 (0%)
Number of people living in household (excluding mother), median (Q1, Q3)	2 (1, 4)	2 (1, 4.2)	0 (0%)
Education level, n (%)			1 (0.03%)
*No school*	178 (6.1%)	4 (5%)	
*P4-P7*	1121 (38.3%)	39 (48.8%)	
*S1-S6*	962 (32.8%)	21 (26.2%)	
*Post-secondary*	668 (22.8%)	16 (20%)	
Occupation with income, n (%)	1460 (49.8%)	36 (45%)	0 (0%)
**Maternal Diagnoses During Pregnancy, n (%)** ^a^			
HIV-infected	309 (10.5%)	5 (6.2%)	0 (0%)
Gestational diabetes	2 (0.1%)	0 (0%)	0 (0%)
Pre-eclampsia or eclampsia	43 (1.5%)	5 (6.2%)	0 (0%)
Gestational hypertension	36 (1.2%)	0 (0%)	0 (0%)
Antepartum hemorrhage	119 (4.1%)	3 (3.8%)	0 (0%)
PPROM	53 (1.8%)	3 (3.8%)	0 (0%)
**Pregnancy and Delivery**			
Sought antenatal care from a doctor/nurse/midwife, n (%)	2924 (99.8%)	80 (100%)	0 (0%)
Number of antenatal care visits, median (Q1, Q3)	4 (4, 5)	4 (4, 5)	1 (0.03%)
*<4 visits*, *n (%)*	699 (23.9%)	18 (22.5%)	
*4–7 visits*, *n (%)*	2123 (72.5%)	61 (76.2%)	
*≥8 visits*, *n (%)*	107 (3.7%)	1 (1.2%)	
Mode of delivery, n (%)			0 (0%)
*Vaginal*	1731 (59.1%)	20 (25%)	
*Assisted vaginal*	31 (1.1%)	1 (1.2%)	
*Caesarean with labour*	971 (33.1%)	48 (60%)	
*Caesarean without labour*	197 (6.7%)	11 (13.8%)	
Delivery at <37 weeks, n (%)	149 (5.1%)	3 (3.8%)	427 (14.57%)
Number of babies delivered, n (%)			0 (0%)
*Individual*	2880 (98.3%)	77 (96.2%)	
*Twin*	48 (1.6%)	3 (3.8%)	
*Triplet*	2 (0.1%)	0 (0%)	
**Newborn Demographics**			
Male sex n (%)	1524 (51.3%)	79 (59.4%)	3 (0.1%)
Birth weight (kg), median (Q1, Q3)	3.2 (2.9, 3.5)	3.2 (2.9, 3.6)	0 (0%)
Length (cm), median (Q1, Q3)	49.6 (48.1, 51)	49.8 (48.3, 51.2)	9 (0.3%)
Apgar at 1 minute, median (Q1, Q3)	9 (8, 9)	9 (8, 9)	0 (0%)
Apgar at 5 minutes, median (Q1, Q3)	10 (10, 10)	10 (10, 10)	0 (0%)

Abbreviations: PPROM = pre-term premature rupture of membranes; Q1 = first quartile; Q3 = third quartile; SD = standard deviation

^a^ Maternal diagnoses during pregnancy were not mutually exclusive

### Post-discharge readmission and mortality

**Fig 2:**
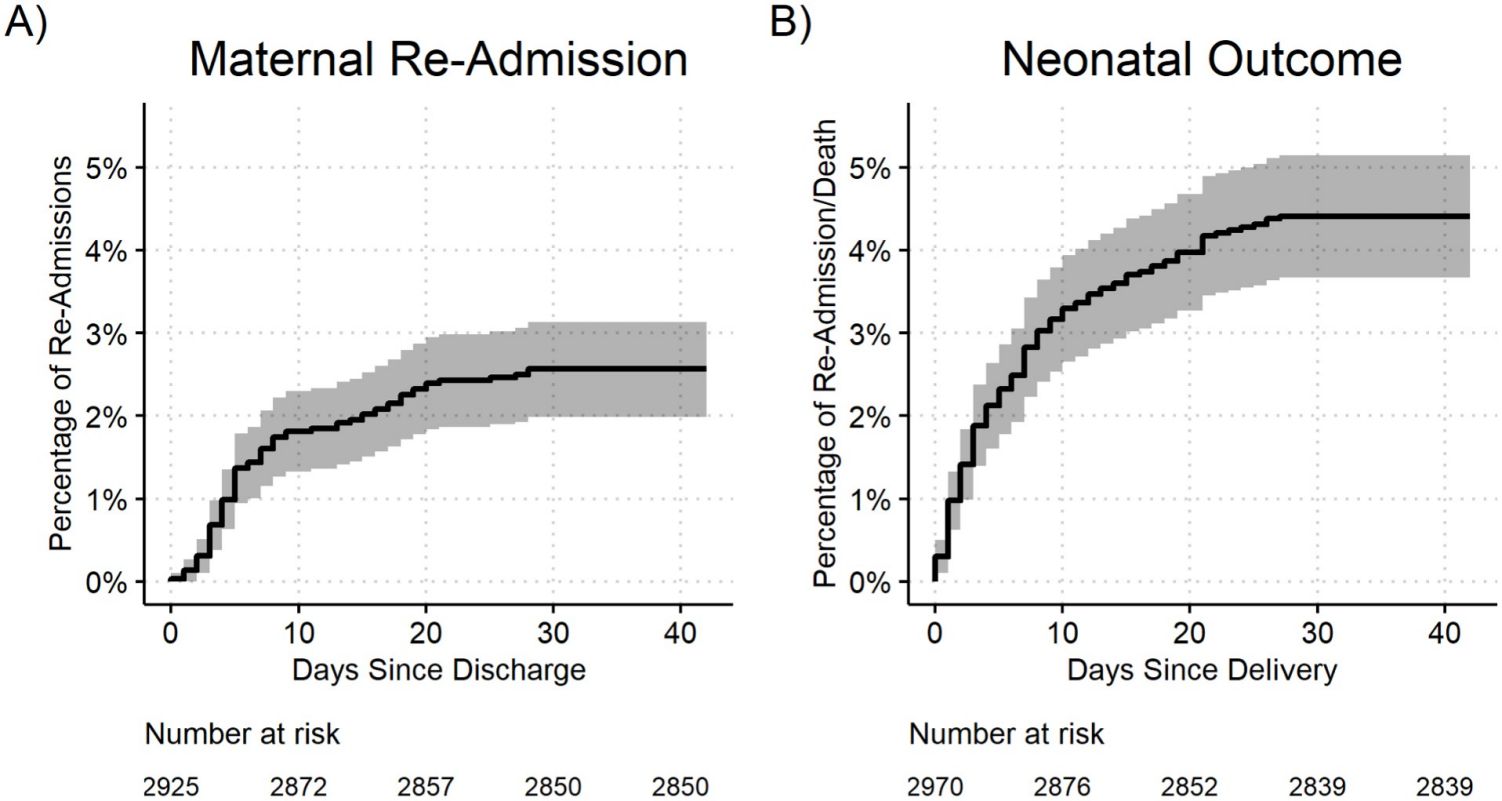
Kaplan-Meier survival plots of time from discharge until A) maternal re-admission and B) newborn re-admission or death. The date of re-admission or death was not known for 5 mothers and 2 neonates and were subsequently excluded from the survival curves.

Over the six-week post-discharge period, 205 (7%) dyads reported at least one outcome of interest (Fig 1). Among postpartum women, there were no deaths reported. Eighty women (2.7%) required re-admission. The medium time to re-admission was five days (3–13.5) in postpartum women with a median length of stay of 5.5 (3–14) days. Among these women, 6 (7.5%) had infants with an adverse outcome within the first 6 weeks as well. In total, 108 infants (3.6%) were re-admitted and 25 (0.8%) died within six weeks of discharge. The median time to readmission from discharge was 3 days (0–9) in newborns (Fig 2). The median time to death among newborns was four (1–14) days after discharge. The relative risk (95% CI) of a neonatal event vs. a maternal event was 1.63 (1.25, 2.15), and for neonatal death vs. neonatal readmission was 0.23 (0.15, 0.36).

Among postpartum women who were readmitted, the most frequently reported diagnoses were surgical site infection (n = 42, 52.5%), puerperal sepsis (n = 35, 43.8%) and malaria (n = 15, 18.8%) (Table 2). These outcomes were not mutually exclusive (22 postpartum women had both surgical site infections and puerperal sepsis). Among infants who were readmitted the most common diagnoses were sepsis (n = 68, 63.0%) and respiratory illness (n = 21, 19.4%). These outcomes were also not mutually exclusive (eight infants had both sepsis and respiratory illnesses).

**Table 2:** Characteristics of postpartum women who were readmitted (n = 80), newborns who were readmitted (n = 108), newborns who died (n = 25) or had either outcome (n = 133).

Characteristics	Readmitted postpartum women (n = 80)	Readmitted newborns (n = 108)	Newborns who died (n = 25)
**Diagnosis During Readmission, n (%)**			
Diarrhea	1 (1.2%)		
Pre-eclampsia or eclampsia	2 (2.5%)		
Heavy bleeding	2 (2.5%)		
HIV-infected	1 (1.2%)		
Malaria	15 (18.8%)		
PPD/psychosis	2 (2.5%)		
Puerperal sepsis	35 (43.8%)		
Other infection	4 (5%)		
Surgical site infection	42 (52.5%)		
Vomiting	1 (1.2%)		
Other	8 (10%)		
Endocrine		2 (1.9%)	0 (0%)
Respiratory distress		21 (19.4%)	2 (8%)
Sepsis		68 (63%)	3 (12%)
Trauma/injury		2 (1.9%)	0 (0%)
**Readmissions**			
Days from delivery to readmission, median (Q1, Q3)	8 (6, 14)	5 (2, 10)	
Days from discharge to readmission, median (Q1, Q3)	5 (3, 13.5)	5 (1, 11)	
**Deaths**			
Days from delivery to death, median (Q1, Q3)			5 (3, 16)
Days from discharge to death, median (Q1, Q3)			4 (1, 14)
Location of death, n (%)			
*Home*			10 (40%)
*Hospital*			11 (44%)
*En route to hospital*			4 (16%)
Hospital care sought prior to death, n (%)			15 (60%)

### Risk factors for adverse outcomes post-discharge

Few factors within the social and demographic domain were associated with increased odds of adverse outcomes in either postpartum women or newborns (Table 3). Number of children in household was associated with a higher probability of maternal readmission (OR: 1.17; 95% CI: 1.03–1.32). Increased time required to reach the hospital for initial delivery was associated with a higher probability of maternal readmission (OR: 1.5; 95% CI: 1.18–1.9) but a lower probability of outcomes in newborns (OR: 0.78; 95% CI: 0.63–0.96). Mothers who used an ambulance to get to the hospital for delivery were also more likely to be readmitted during the postnatal period (OR: 2.11; 95% CI: 1.06–3.98).

**Table 3:** Odds ratios for maternal readmission and newborn readmission and mortality from univariable and multivariable models for variables in the social and demographic factors domain.

Term (Reference Group)	N (%), Mean (SD), or Median (Q1, Q3)		Maternal Outcome		Newborn Outcome	
		N Missing (%)	Univariable OR	Multivariable OR	Univariable OR	Multivariable OR
Married	2751 (93.9%)	0 (0%)	0.98 (0.43, 2.81)	1.13 (0.35, 3.93)	1.33 (0.63, 3.43)	0.97 (0.34, 2.98)
Lives with father	2572 (87.8%)	0 (0%)	0.87 (0.47, 1.75)	0.8 (0.38, 2)	1.35 (0.77, 2.6)	1.37 (0.68, 3.18)
Number of children in household	2 (1, 3)	0 (0%)	**1.16 (1.02, 1.29)**	**1.17 (1.03, 1.32)**	0.92 (0.82, 1.04)	0.94 (0.82, 1.06)
Employs a domestic worker in the home	303 (10.3%)	0 (0%)	0.45 (0.14, 1.09)	0.42 (0.13, 1.04)	1.17 (0.65, 1.97)	1.21 (0.66, 2.1)
Anyone smokes tobacco at home	130 (4.4%)	1 (0.03%)	1.78 (0.68, 3.85)	1.38 (0.52, 3.09)	0.88 (0.31, 1.97)	1.01 (0.35, 2.32)
Used herbs during pregnancy	2249 (76.8%)	0 (0%)	1.32 (0.77, 2.41)	1.12 (0.62, 2.13)	1.07 (0.71, 1.67)	1.13 (0.72, 1.83)
Sought care from traditional birth attendant	866 (29.6%)	0 (0%)	1.37 (0.85, 2.16)	1.27 (0.76, 2.08)	1.06 (0.71, 1.54)	1.03 (0.67, 1.55)
Education level ^a^	3 (2, 3)	1 (0.03%)	0.86 (0.67, 1.11)	1.15 (0.85, 1.55)	1.09 (0.89, 1.33)	0.95 (0.76, 1.2)
Paid occupation outside home	1460 (49.8%)	0 (0%)	0.82 (0.52, 1.28)	1.06 (0.64, 1.75)	1.29 (0.9, 1.85)	1.17 (0.79, 1.72)
Transport time ^b^	2 (1, 3)	0 (0%)	**1.54 (1.24, 1.91)**	**1.5 (1.18, 1.9)**	**0.78 (0.64, 0.94)**	**0.78 (0.63, 0.96)**
Transport mode (Motor transport)	2630 (89.8%)	0 (0%)				
*Non-motor transport*	76 (2.6%)		1.06 (0.17, 3.49)	1.23 (0.2, 4.13)	1.6 (0.55, 3.66)	1.44 (0.5, 3.34)
*Ambulance*	224 (7.6%)		**2.43 (1.26, 4.33)**	**2.12 (1.06, 3.98)**	1.17 (0.59, 2.11)	1.52 (0.73, 2.9)
Someone other than mother decided to go hospital for delivery	714 (24.4%)	0 (0%)	0.9 (0.51, 1.5)	0.65 (0.36, 1.11)	0.79 (0.5, 1.21)	0.85 (0.53, 1.34)

^a^ Analysed as continuous variable (1 = no school; 2 = P4-P7; 3 = S1-S6; 4 = Post secondary)

^b^ Analysed as continuous variable (1 = <30 minutes; 2 = 30–60 minutes; 3 = 1–2 hours; 4 = >2 hours)

Within the pregnancy and antenatal history domain, parity was significantly associated with maternal outcomes in the multivariable analysis. None of the variables grouped within this domain were statistically significant in relation to neonatal outcomes (Table 4).

**Table 4:** Odds ratios for maternal readmission and newborn readmission and mortality from univariable and multivariable models for variables in the pregnancy history and antenatal care domain.

Term (Reference Group)	N (%), Mean (SD), or Median (Q1, Q3)	N Missing (%)	Maternal Outcome		Newborn Outcome	
			Univariable OR	Multivariable OR	Univariable OR	Multivariable OR
Due date known	2508 (85.6%)	0 (0%)	0.95 (0.53, 1.86)	0.98 (0.54, 1.94)	1.27 (0.76, 2.28)	1.14 (0.67, 2.06)
Maternal age (18–35 years)	2657 (90.7%)	4 (0.14%)				
*<18 years*	79 (2.7%)		0.93 (0.15, 3.04)	1.18 (0.19, 3.97)	0.54 (0.09, 1.76)	0.57 (0.09, 1.87)
*>35 years*	190 (6.5%)		1.16 (0.45, 2.5)	0.77 (0.26, 1.91)	0.45 (0.14, 1.08)	0.48 (0.14, 1.28)
Parity	2 (1, 3)	0 (0%)	**1.13 (1, 1.27)**	**1.16 (1, 1.32)**	0.95 (0.84, 1.05)	0.98 (0.86, 1.11)
Prenatal vitamins during pregnancy	2826 (96.5%)	0 (0%)	2.94 (0.64, 52.07)	2.65 (0.57, 47.1)	1.53 (0.57, 6.28)	1.32 (0.48, 5.45)
History of chronic health conditions before pregnancy ^a^	2529 (86.3%)	0 (0%)	0.9 (0.43, 1.68)	1.37 (0.53, 3.02)	1.19 (0.71, 1.9)	1.74 (0.88, 3.2)
HIV-infected	309 (10.5%)	0 (0%)	0.56 (0.19, 1.26)	0.41 (0.12, 1.3)	0.71 (0.35, 1.31)	0.47 (0.19, 1.1)
Malaria diagnosis during pregnancy	331 (11.3%)	0 (0%)	1.55 (0.81, 2.74)	1.34 (0.65, 2.62)	0.66 (0.32, 1.21)	0.55 (0.25, 1.09)
Other pre-pregnancy complications ^b^	251 (8.6%)	0 (0%)	1.74 (0.86, 3.2)	1.52 (0.7, 3.05)	1.01 (0.51, 1.82)	0.94 (0.44, 1.83)
Urinary tract infection during pregnancy	892 (30.4%)	0 (0%)	**1.72 (1.09, 2.69)**	1.52 (0.75, 3.23)	1.31 (0.9, 1.89)	1.46 (0.78, 2.76)
Other pre-pregnancy infections	256 (8.7%)	0 (0%)	0.69 (0.24, 1.56)	0.7 (0.23, 1.79)	1.66 (0.95, 2.73)	1.69 (0.83, 3.26)
Previous admission	262 (8.9%)	1 (0.03%)	1.13 (0.5, 2.24)	0.9 (0.38, 1.9)	1.49 (0.84, 2.48)	1.59 (0.86, 2.79)
Diagnosed with condition during pregnancy ^c^	1441 (49.2%)	0 (0%)	1.49 (0.95, 2.35)	1.03 (0.43, 2.37)	1.2 (0.84, 1.72)	0.88 (0.43, 1.78)
Number of antenatal care visits	4 (4, 5)	1 (0.03%)	0.98 (0.84, 1.14)	0.98 (0.83, 1.14)	**1.13 (1.01, 1.27)**	1.11 (0.98, 1.25)

^a^ Includes diabetes, chronic hypertension, other heart disease, kidney disease, HIV, sickle cell, hepatitis B/C, tuberculosis, chronic mental illness, or other chronic health conditions (free text option)

^b^ Includes gestational diabetes, pre-eclampsia, eclampsia, gestational hypertension, antepartum hemorrhage/vaginal bleeding, PPROM, or preterm labour.

^c^ Includes gestational diabetes, pre-eclampsia, eclampsia, gestational hypertension, antepartum hemorrhage/ vaginal bleeding, PPROM, preterm labour, malaria, HIV, urinary tract infection, tuberculosis, anemia, or other infections (free text option).

The delivery domain was the most important category for maternal outcomes (Table 5). Mode of delivery was a key, and highly prevalent risk factor in this category. Postpartum women who delivered via caesarean had more than double the odds of a post-discharge adverse outcome than those who delivered vaginally (OR: 2.92; 95% CI: 1.51–6.06). In addition, the detection of meconium in the amniotic fluid was associated with an increase in the odds of a post-discharge adverse outcome (OR: 1.9; 95% CI: 1.14–3.1). Interestingly, postpartum women reporting increasing numbers of vaginal exams during labour had increased odds of both maternal (OR: 1.15; 95%CI: 1.02–1.28) and newborn outcomes (OR: 1.11; 95%CI: 1.01–1.22).

**Table 5:** Odds ratios for maternal readmission and newborn readmission and mortality from univariable and multivariable models for variables in the delivery domain. Birthweight was analysed as both a continuous and categorical variable with the continuous version being used in the multivariable model in the odds ratios presented for the other variables.

Term (Reference Group)	N (%), Mean (SD), or Median (Q1, Q3)	N Missing	Maternal Outcome		Newborn Outcome	
			Univariable OR	Multivariable OR	Univariable OR	Multivariable OR
Antibiotics during admission	124 (4.2%)	5 (0.17%)	**2.51 (1.1, 5.03)**	1.75 (0.74, 3.66)	0.9 (0.31, 2.02)	0.84 (0.29, 1.93)
Episiotomy	517 (17.6%)	4 (0.14%)	**0.23 (0.07, 0.57)**	0.5 (0.14, 1.39)	1.32 (0.84, 2)	1.38 (0.83, 2.26)
Degree of tearing (None)	2465 (84.1%)	30 (1.02%)				
*1*	280 (9.6%)		0.58 (0.2, 1.31)	1.67 (0.52, 4.63)	0.69 (0.32, 1.31)	0.74 (0.33, 1.48)
*2 or more*	155 (5.3%)		0.4 (0.07, 1.3)	1.15 (0.18, 4.21)	0.96 (0.4, 1.94)	0.93 (0.38, 1.94)
Labour induced	108 (3.7%)	1 (0.03%)	1 (0.24, 2.72)	1.23 (0.28, 3.59)	0.83 (0.25, 2.03)	0.72 (0.22, 1.8)
Labour obstructed	280 (9.6%)	108 (3.69%)	**3.52 (2.12, 5.68)**	1.61 (0.91, 2.81)	0.98 (0.53, 1.67)	0.86 (0.44, 1.56)
Meconium in amniotic fluid	519 (17.7%)	142 (4.85%)	**2.66 (1.67, 4.17)**	**1.9 (1.14, 3.1)**	1.35 (0.88, 2.03)	1.34 (0.86, 2.05)
Number of vaginal exams	2 (1, 3)	3 (0.1%)	**1.13 (1.01, 1.25)**	**1.15 (1.02, 1.28)**	1.09 (0.99, 1.19)	**1.11 (1.01, 1.22)**
Caesarean delivery	1168 (39.9%)	0 (0%)	**4.55 (2.8, 7.68)**	**2.92 (1.51, 6.06)**	1.09 (0.76, 1.55)	1.18 (0.74, 1.92)
More than one baby delivered	50 (1.7%)	0 (0%)	2.11 (0.73, 4.86)	2.83 (0.89, 7.42)	1.88 (0.78, 3.88)	1.9 (0.74, 4.21)
Time from admission till delivery, per day	0.3 (0.1, 0.7)	6 (0.2%)	1.02 (0.87, 1.09)	0.98 (0.77, 1.11)	1.02 (0.91, 1.09)	1.01 (0.89, 1.12)
Time from admission till discharge, per day	1.6 (1, 3.1)	4 (0.14%)	**1.05 (1.01, 1.08)**	1.02 (0.93, 1.06)	1.02 (0.97, 1.05)	1.01 (0.93, 1.06)
Time from labour till delivery, per day	0.9 (0.5, 1.6)	258 (8.81%)	1.05 (0.96, 1.11)	1.02 (0.9, 1.09)	0.89 (0.73, 1.03)	0.82 (0.65, 0.99)
Baby resuscitated after birth	496 (16.7%)	373 (12.55%)	1.45 (0.85, 2.37)	1.49 (0.82, 2.62)	1.24 (0.79, 1.88)	1.12 (0.69, 1.74)
Apgar score at 1 minute	9 (8, 9)	0 (0%)	0.85 (0.69, 1.07)	1.24 (0.88, 1.8)	0.84 (0.71, 1.01)	1 (0.77, 1.31)
Apgar score at 5 minutes	10 (10, 10)	0 (0%)	**0.67 (0.52, 0.92)**	0.64 (0.39, 1.06)	**0.73 (0.58, 0.95)**	0.77 (0.52, 1.15)
Visit was referred to MRRH from another health centre	1082 (36.9%)	0 (0%)	**2.46 (1.58, 3.87)**	**1.93 (1.21, 3.11)**	0.94 (0.65, 1.35)	0.89 (0.6, 1.31)
Delivery ≤37 weeks	149 (5.1%)	427 (14.57%)	0.53 (0.13, 1.44)	0.4 (0.09, 1.25)	0.94 (0.42, 1.83)	0.87 (0.36, 1.78)
Birthweight of baby, per kg	3.2 (0.5)	0 (0%)	1.24 (0.79, 1.94)	1.03 (0.63, 1.67)	0.97 (0.67, 1.4)	1.05 (0.7, 1.56)
Birthweight of baby ≤2.5 kg	232 (7.8%)	0 (0%)	1.11 (0.46, 2.28)	1.11 (0.4, 2.66)	1.48 (0.8, 2.53)	1.37 (0.69, 2.53)

To co-examine factors influencing the mother-infant pair, maternal and newborn discharge factors collected on all dyads were assessed in relation to both maternal and newborn outcomes respectively. Among maternal discharge factors (Table 6), only a higher maternal heart rate measured at the time of hospital discharge was significantly associated with maternal readmission (OR 1.02; 95% CI: 1.01–1.03).

**Table 6:** Odds ratios for maternal readmission and newborn readmission and mortality from univariable and multivariable models for variables in the maternal discharge domain. Delivery mode (from the delivery domain) was included as an additional covariate but is not reported in this table. MUAC was analysed as both a continuous and categorical variable with the continuous version being used in the multivariable model in the odds ratios presented for the other variables.

Term (Reference Group)	N (%), Mean (SD), or Median (Q1, Q3)	N Missing (%)	Maternal Outcome		Newborn Outcome	
			Univariable OR	Multivariable OR	Univariable OR	Multivariable OR
Maternal systolic blood pressure	112.4 (12.5)	0 (0%)	**0.98 (0.96, 1)**	0.98 (0.95, 1.01)	1.01 (0.99, 1.02)	0.99 (0.97, 1.01)
Maternal diastolic blood pressure	70.6 (10)	0 (0%)	0.99 (0.97, 1.01)	1.02 (0.99, 1.05)	**1.02 (1, 1.03)**	**1.03 (1, 1.06)**
Maternal hypertension at discharge	132 (4.5%)	0 (0%)	0.82 (0.2, 2.23)	0.89 (0.19, 3.13)	1.45 (0.64, 2.86)	0.83 (0.31, 2.04)
Maternal temporal artery temperature, per degree	36.5 (0.5)	0 (0%)	1.1 (0.69, 1.73)	1.27 (0.79, 2.02)	1.14 (0.79, 1.64)	1.1 (0.75, 1.61)
Maternal symptoms present ^a^	255 (8.7%)	0 (0%)	1.52 (0.73, 2.84)	1 (0.47, 1.91)	**1.9 (1.12, 3.08)**	**1.76 (1.02, 2.91)**
Able to start breastfeeding	2907 (99.2%)	0 (0%)	0.62 (0.13, 11.09)	0.51 (0.1, 9.47)	1 (0.21, 17.94)	1.03 (0.21, 18.55)
Maternal best SpO2	97.9 (1.3)	0 (0%)	0.97 (0.82, 1.15)	1.02 (0.86, 1.22)	0.94 (0.82, 1.07)	0.92 (0.81, 1.06)
Maternal best heart rate	96.9 (16.4)	0 (0%)	**1.01 (1, 1.03)**	**1.02 (1, 1.03)**	1 (0.99, 1.01)	0.99 (0.98, 1.01)
Maternal respiratory rate	20.8 (3.2)	0 (0%)	0.94 (0.87, 1.01)	0.95 (0.88, 1.02)	1.04 (0.98, 1.09)	1.04 (0.99, 1.1)
Maternal haemoglobin, per g/dL	11.3 (2)	1 (0.03%)	**0.82 (0.73, 0.91)**	0.9 (0.8, 1.01)	**0.91 (0.83, 0.99)**	**0.9 (0.82, 0.99)**
Maternal random glucose	5.8 (1.8)	1 (0.03%)	1.05 (0.92, 1.17)	1.06 (0.93, 1.2)	1.05 (0.95, 1.15)	1.07 (0.96, 1.17)
MUAC, per mm	279.9 (34.8)	1 (0.03%)	1 (1, 1.01)	1 (1, 1.01)	1 (1, 1.01)	1 (1, 1.01)
*Maternal malnutrition*, *MUAC <230mm*	112 (3.8%)	1 (0.03%)	0.32 (0.02, 1.45)	0.24 (0.01, 1.11)	0.6 (0.15, 1.63)	0.57 (0.14, 1.57)
Time from delivery till discharge, per day	1 (0.6, 2.6)	9 (0.31%)	**1.05 (1, 1.09)**	1 (0.9, 1.05)	1.02 (0.95, 1.06)	1.01 (0.93, 1.06)

^a^ Includes headache, visual changes, chest pain, shortness of breath, nausea with vomiting, abdominal pain on the right side, foul smelling vaginal discharge, stiff neck, or cough.

Maternal clinical variables (Table 6) including an elevated maternal diastolic blood pressure, lower hemoglobin and postpartum women who had symptoms present at discharge (including headache, visual changes, chest pain, shortness of breath, nausea with vomiting, abdominal pain on the right side, stiff neck, cough, foul smelling vaginal discharge) were associated with newborn post-discharge outcomes. Among newborn discharge variables (Table 7), higher neonatal heart rate at discharge (OR: 1.94; 95%CI: 1.19–3.09), temperature (OR: 1.66; 95% CI: 1.28–2.13) measured at the time of hospital discharge, and male sex (OR: 1.48; 95% CI: 1.02–2.15) were all significantly associated with neonate death or readmission, in the adjusted analysis.

**Table 7:** Odds ratios for maternal readmission and newborn readmission and mortality from univariable and multivariable models for variables in the newborn discharge domain. Delivery mode (from the delivery domains) was included as an additional covariate but is not reported in this table. Newborn temperature and SpO2 were analysed as both a continuous and categorical variable with the continuous version being used in the multivariable model in the odds ratios presented for the other variables.

Term (Reference)	N (%), Mean (SD), or Median (Q1, Q3)	N Missing (%)	Maternal Outcome		Newborn Outcome	
			Univariable OR	Multivariable OR	Univariable OR	Multivariable OR
Male baby	1524 (51.3%)	3 (0.1%)	0.9 (0.58, 1.39)	0.87 (0.55, 1.38)	**1.47 (1.03, 2.12)**	**1.48 (1.02, 2.15)**
Newborn defecated	2668 (89.8%)	3 (0.1%)	1.35 (0.63, 3.49)	1.55 (0.61, 4.56)	0.68 (0.41, 1.19)	0.54 (0.28, 1.07)
Newborn made urine	2575 (86.6%)	4 (0.13%)	0.76 (0.42, 1.48)	**0.43 (0.21, 0.97)**	1 (0.59, 1.8)	1.22 (0.63, 2.51)
Newborn length, per cm	49.3 (2.4)	9 (0.3%)	1.06 (0.97, 1.17)	1.05 (0.94, 1.18)	0.99 (0.92, 1.07)	0.95 (0.89, 1.03)
Newborn head circumference, per cm	35.1 (1.4)	8 (0.27%)	1.05 (0.9, 1.21)	0.94 (0.76, 1.16)	1.1 (0.98, 1.23)	1.09 (0.95, 1.24)
Newborn weight, per kg	3.1 (0.5)	10 (0.34%)	1.15 (0.71, 1.84)	0.97 (0.44, 2.09)	0.99 (0.67, 1.44)	0.72 (0.4, 1.29)
Newborn MUAC, per mm	102 (9.8)	21 (0.71%)	1.01 (0.99, 1.03)	1.01 (0.98, 1.04)	1.01 (0.99, 1.03)	1.02 (0.99, 1.05)
Newborn respiratory rate	53.9 (11.5)	7 (0.24%)	1.01 (0.99, 1.03)	1.01 (0.99, 1.03)	**1.02 (1, 1.03)**	1 (0.99, 1.02)
Newborn blood glucose	3.7 (0.9)	9 (0.3%)	0.99 (0.77, 1.25)	0.93 (0.71, 1.2)	0.92 (0.74, 1.12)	0.88 (0.71, 1.07)
Newborn complications present ^a^	62 (2.1%)	0 (0%)	1.95 (0.58, 4.84)	1.85 (0.54, 4.74)	1.87 (0.72, 4.06)	1.79 (0.67, 3.97)
Newborn temporal artery temperature, per degree	36.9 (0.6)	7 (0.24%)	1.21 (0.87, 1.63)	1 (0.69, 1.41)	**1.77 (1.42, 2.19)**	**1.66 (1.28, 2.13)**
*Normal*, *36*.*5 to 37*.*5°C (reference)*	1853 (62.3%)					
*Hypothermic*, *<36*.*5°C*	738 (24.8%)		0.6 (0.31, 1.07)	0.67 (0.34, 1.21)	0.82 (0.5, 1.29)	0.83 (0.51, 1.31)
*Fever*, *>37*.*5°C*	374 (12.6%)		1.4 (0.75, 2.44)	0.83 (0.5, 1.33)	**2.29 (1.47, 3.5)**	1.13 (0.78, 1.63)
Weight change at discharge from birth, per kg	-0.1 (0.2)	10 (0.34%)	0.58 (0.21, 1.75)	1.27 (0.42, 3.99)	1.1 (0.45, 2.73)	1.19 (0.47, 3)
SpO_2_, %	95.5 (94, 97)	7 (0.24%)	1.06 (0.98, 1.16)	1.03 (0.95, 1.12)	0.95 (0.91, 1.01)	0.95 (0.9, 1.01)
Hypoxia, SpO_2_ < 95%	1056 (35.5%)	7 (0.24%)	0.74 (0.45, 1.19)	1 (0.99, 1.02)	1.15 (0.8, 1.65)	**1.01 (1, 1.02)**
Newborn best heart rate	135.4 (16.2)	7 (0.24%)	1.01 (0.99, 1.02)	1.13 (0.59, 2.08)	**1.02 (1, 1.03)**	**1.94 (1.2, 3.09)**

^a^ Includes increased or decreased tone, restlessness, irritability, or lethargy at assessment, bulging fontanelle, jaundice, abdominal distension.

### Postnatal care visits

**Fig 3:**
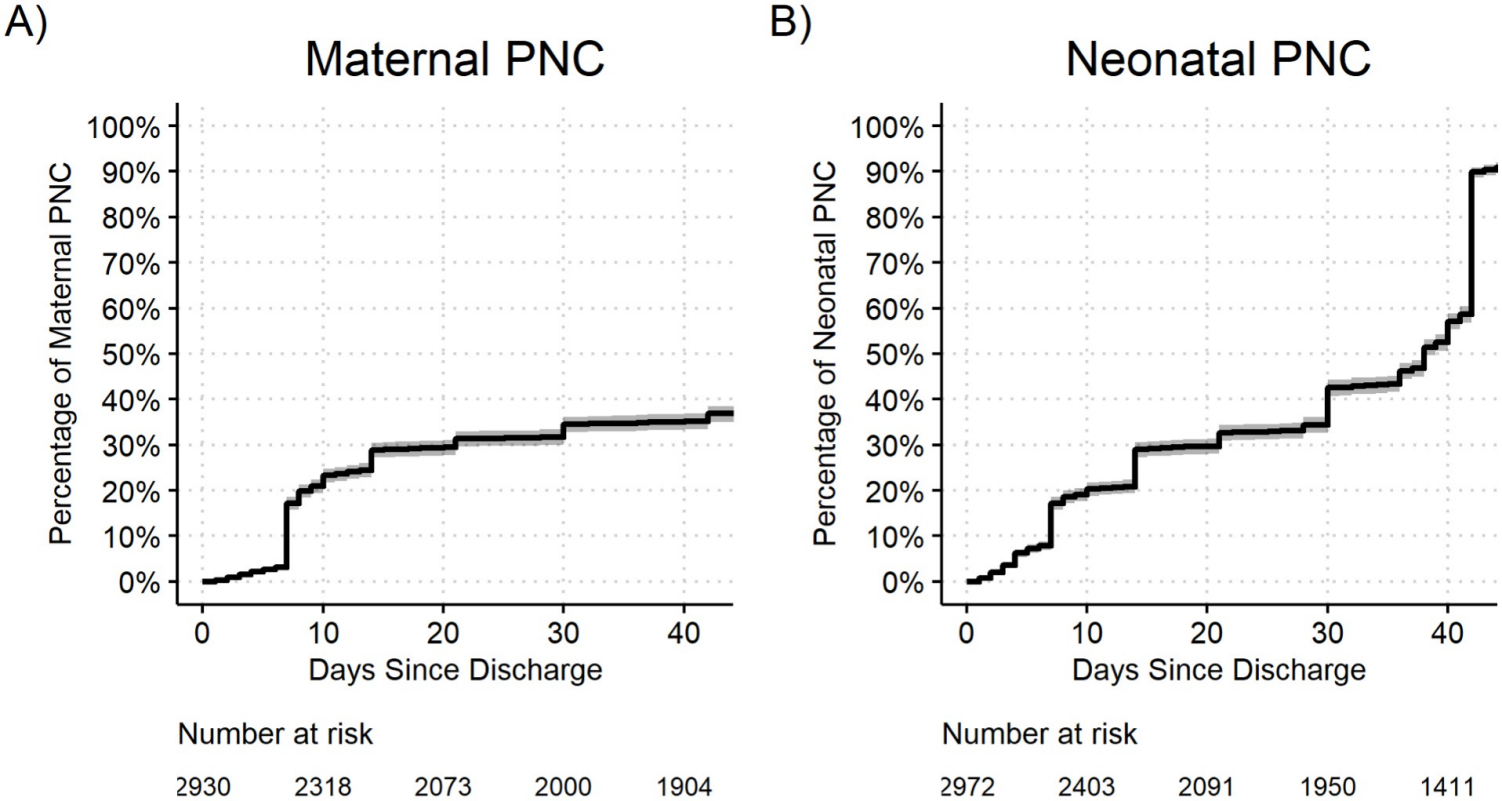
Kaplan-Meier survival plots of time from discharge until A) first maternal post-natal care visit and B) first newborn post-natal care visit.

Amongst the 2930 dyads, 1130 (38.6%) postpartum women reported completing at least one postnatal care visit for themselves, though only 83 (2.8%) completed all three WHO-recommended postnatal care visits in the first six weeks. The median time (IQR) to the first visit was 8 (7–14) days following birth (Fig 3). A total of 2769 (93.2%) postpartum women reported receiving at least one postnatal assessment for their newborn, though these occurred substantially later than for themselves, with a median time to the first assessment of 36 (14–42) days, many of which coincided with the first immunization at 42 days and only 266 (9.0%) received three or more visits (Table 8).

**Table 8:** Postnatal care-seeking of dyads discharged together with eligible follow-up (n = 2930 mothers and 2972 newborns).

Characteristics	Mothers (n = 2930)	Newborns (n = 2972)	N Missing (%)
Sought care for themselves after being discharged with newborn post-delivery, n (%)	1130 (38.6%)		9 (0.31%)
Number of routine postpartum visits, n (%)			
*0*	1833 (62.6%)		
*1*	759 (25.9%)		
*2*	248 (8.5%)		
*≥3*	83 (2.8%)		
Number of days from birth to first postnatal care visit, median (Q1, Q3)	8 (7, 14)		
Sought care for the newborn at a facility after being home post delivery, n (%)		2769 (93.2%)	34 (1.14%)
Number of routine newborn check ups after leaving hospital following birth, n (%)			37 (1.24%)
*0*		198 (6.7%)	
*1*		1380 (46.4%)	
*2*		1091 (36.7%)	
*≥3*		266 (9%)	
Number of days from birth to first postnatal check up, median (Q1, Q3)		36 (14, 42)	234 (7.87%)

Factors associated with receiving any postnatal care visits for both postpartum women and newborns included caesarean deliveries, referrals, and women who had more antenatal care visits (S4 Table). Postpartum women who were diagnosed with a condition (gestational diabetes, pre-eclampsia, eclampsia, gestational hypertension, antepartum hemorrhage, preterm premature rupture of membranes, preterm labour, malaria, HIV, urinary tract infection, tuberculosis, anemia or other infections) during pregnancy, who sought care from a traditional birth attendant and those who travelled by ambulance to the hospital for delivery were also more likely to receive postnatal care. Newborns whose mothers used herbal remedies during pregnancy were less likely to receive postnatal care. Families where someone other than the mother made the decision to go to the hospital for delivery were also less likely to receive postnatal care for their infants (S2 Table). Women older than 35 years of age were less likely to receive postnatal care (S3 Table).

## Discussion

This study addresses an important but neglected aspect of postpartum epidemiology by investigating demographic and clinical risk factors for mortality and re-admission in both mothers and newborns in the first six weeks following routine delivery and discharge within a resource-limited setting in southwestern Uganda. In this exploratory study, one in 14 dyads (7%) in which both mother and newborn were deemed fit for discharge, experienced a readmission or death within six weeks of discharge following delivery. Among our cohort of interest, the observed newborn mortality and readmission rate of 0.8% and 3.6%, respectively indicates a significant level of risk even among those who were deemed well enough to be discharged with their mothers. Importantly, the re-admission rate was almost certainly an underestimation of the true rate of severe illness post-discharge, since only 50% of the newborn deaths occurred during a subsequent readmission. This potentially suggests additional episodes of severe illness that did not result in death or care seeking occurred during follow up.

Within our five pre-specified domains of analysis (social and demographic, pregnancy history and antenatal care, delivery, maternal discharge, and neonatal discharge), the delivery domain included the most significant variables associated with post-discharge outcomes among postpartum women suggesting that both surgical and otherwise complicated deliveries are primary contributors to post-discharge risk. Caesarean rates at regional referral facilities in Uganda are more likely to be high (>25%) compared to lower level facilities, though the Mbarara Regional Referral Hospital (MRRH) has reported rates exceeding 40% in previous studies, in line with our present findings [11, 32, 40, 41]. Caesarean delivery as an independent risk factor for maternal readmission is well documented, and the finding that more than 50% of maternal readmissions were related to this suggests the need to improve monitoring and follow-up among this group of women [11, 42–44]. At discharge, however, when controlling for mode of delivery, neither vital signs, nor the presence of key symptoms or warning signs, predicted post-discharge readmission among postpartum women. Among newborns however, both maternal and newborn clinical discharge characteristics were associated with post-discharge neonatal death or readmission. Maternal anemia at discharge was associated with risk for infants in the postnatal period. Further, a higher number of vaginal exams during delivery was identified as a risk factor in both postpartum women and newborns in this study. Vaginal exams have previously been correlated with febrile morbidities and postpartum infections in both mothers and newborns [45–47]. Together, this evidence underscores the importance of considering the dyadic unit in care and detecting and treating infections early. This is substantiated by existing studies in sub-Saharan Africa which posit that maternal complications impair newborns’ ability to survive and thrive in the postnatal period [14, 16, 48].

Among postpartum women, surgical site infection, puerperal sepsis and malaria made up most of the reported diagnoses, though these were not mutually exclusive. High rates of postpartum infection in this population have been previously reported and is in line with these findings [11]. The timing and clinical presentation of post-discharge malaria infections may partially account for the poor association between maternal and newborn discharge variables and post-discharge maternal outcomes as malaria infection is a non-obstetric related cause of readmission. Existing literature suggests postpartum women have a higher risk of malaria infection than those pre-pregnancy and lower risk than pregnant women, though notably, evidence is sparse and conflicting and further epidemiologic investigations are needed in this area [49, 50]. Although we did not capture factors related to the three-delays model for care seeking during the post-discharge period, we did identify that a type II delay in care (transport time to the facility) prior to facility delivery was an important risk factor for maternal post-discharge outcomes.

In newborns, sepsis and respiratory infections were the most common causes of readmission and death, consistent with existing literature [5, 17, 22]. However, in our study, this data was collected by maternal self-report and could potentially include failure to thrive or other conditions due to the absence of blood cultures and the non-specific clinical presentation of sepsis in neonates [51–53]. The timing of newborn outcomes together with the clinically significant discharge variables indicate infection may have developed prior to hospital discharge. That approximately 25% of newborn readmissions occurred on the same day as discharge suggests more robust discharge criteria, longer duration of stay or close post discharge follow-up may avert some readmissions.

Contrary to our expectations, newborn characteristics such as birthweight or gestational age were not associated with post-discharge neonatal outcomes. However, as this study excluded newborns who were admitted directly to the neonatal unit following birth, the effects of prematurity and low birthweight were not expected to be as significant in this analysis as have been reported elsewhere [54–57]. Our results showed that postpartum women who had delivered vaginally were often discharged with their newborns before the recommended 24-hour observation period. Despite significant disparities in the definition of early discharge, previous studies looking at postnatal complications among healthy dyads who were discharged early have indicated that this is still a risk factor for adverse newborn outcomes. Notably, there is a dearth of literature focussed on this critical period and population within low resource settings to inform contextually appropriate care guidelines [19, 25]. However, given the observed timing of newborn deaths and readmissions in our study, longer initial hospital stays for at-risk newborns -potentially identified using maternal and newborn clinical discharge variables, could provide an opportunity to detect and treat infections early.

The results of this study highlight that both maternal and newborn adverse outcomes generally occurred within the first two weeks of discharge. However, standard postnatal care visits in these groups often occurred much later than two weeks. This delay in care is especially concerning for newborns where 75% of readmissions or deaths occurred within the first six days after discharge. Notably, only 2.8% of total dyads enrolled completed the recommended the number of maternal-child visits within the six-week postnatal period. Previous studies using national demographic health data in Uganda have found that between 10.7–50% of postpartum women and 22.5–41% newborns attend at least one postnatal care visit while postnatal care rates in Mbarara are reported between 4–7% [58–60]. Although our data only represents dyads in the southwestern region of Uganda, the 93% postnatal care attendance captured for newborns by the end of the six-week period is likely due to vaccination visits. Maternal self-report may have conflated the six-week family planning and vaccination visit with the routine postnatal care or a wellness checks spaced throughout the postnatal period, which likely inflated our newborn postnatal care rate. The range of data in our study and others emphasises current gaps in care coverage, surveillance of care continuum participation and understanding of facilitators and barriers to care in this period. Postnatal care includes screening for illness in both the mother and newborn using clinical signs, referral, when necessary, promotion of breastfeeding and counselling on family planning, nutrition, hygiene and mental health. Many of the clinical variables used to routinely screen for illness at these visits were significant discharge variables captured in our study. This, together with the timing of postnatal outcomes in our study suggests that early postnatal care assessments, especially for vulnerable dyads are crucial for disease detection and early treatment initiation to reduce severe infections during the postnatal period [61].

The timing of post-discharge outcomes observed in both mothers and newborns suggest that a general one-size-fits-all approach to postnatal care, is insufficient to detect and prevent many instances of severe illness or death among vulnerable dyads. Furthermore, this approach places an undue burden on already strained health systems and families for follow-up care when the majority could be safely discharged with minimal follow-up. Efforts to develop evidence-based approaches to post-partum care using a risk-based (patient centered) model of care within low-resourced settings is imperative. Our postnatal care results indicated that maternal knowledge of risk informed health-seeking in the postnatal period. This could be leveraged for targeted discharge counselling opportunities or to inform risk-based follow-up care for the most vulnerable dyads to prevent, detect and treat infections early to reduce the incidence of severe illness. Similar approaches have been suggested in the context of post-discharge follow-up for pediatric sepsis in resource limited settings [62]. Overall, these results suggest that maternal delivery characteristics and newborn discharge characteristics are most likely to provide key variables for future patient-centered, risk-based models of care for mother-newborn dyads.

In this study we identified that postpartum women who were engaged in the health system through antenatal care, or due to high-risk deliveries (e.g., caesarean, referral or underlying condition) continued to pursue care during the postnatal period. This is a positive finding and suggests that knowledge of risk influences postnatal care–corroborated by previous studies [32]. Furthermore, this suggests that a more patient-centered approach to care is more likely to produce adherence to prescribed follow-up compared to general recommendations that are not individualized.

This study has several limitations. First, the data were collected from one regional referral facility in Southwestern Uganda and may not be generalizable to other contexts, particularly those with lower rates of caesarean deliveries. Although caesareans were a strong risk factor for maternal readmissions, further data are needed to distinguish whether this was related to the caesarean itself or the underlying condition that necessitated it. Socioeconomic status has a direct impact on the ability to seek care but studying these variables in LMIC settings is difficult as income, wealth, and consumption indicators used in high income countries do not always translate well to LMIC settings. Thus, although many of the socioeconomic variables in our study were not significant, this requires further study and different approaches including out of pocket expenditure. In addition, future studies identifying facility related practices and the discharge process are needed. Our outcomes were broadly classified as either maternal or newborn. Many outcomes occurred at home, without an interaction with a healthcare worker. Thus, a more granular assessment of outcomes was not completed, and self-report of outcomes and their possible causes may have been biased or mis-reported. Further, while maternal outcomes only included readmission (no maternal deaths were recorded), neonatal outcomes included both re-admission and deaths. This may be important as different outcomes may be associated with different sets of predictors. For example, predictors of outcomes related to postpartum pre-eclampsia and sepsis are likely to have non-overlapping elements. Similarly, early and late onset neonatal sepsis, failure to thrive, and non-infectious outcomes (e.g., congenital abnormalities) may suffer from similar limitations. Though our overall adverse outcome rate of 7% is high, it was not sufficiently powered to further sub-setting outcomes based on such considerations. Future studies aiming to develop clinical prediction algorithms for postnatal outcomes must consider the need for robust outcome definitions with adequate sample sizes for such analyses. Approximately 5% of the dyads that were discharged together (161 post-partum women, 162 newborns) had unsuccessful follow-up, although the characteristics of those with unsuccessful follow-up were broadly similar to the analyzed cohort. Regardless, there may be other undocumented differences that may bias the likelihood of maternal or neonatal outcome. Finally, the high number of analyses may have contributed to identification of association by chance alone, rather than due to a true association. Thus, results with marginal significance should be interpreted within this light. However, the comprehensive nature of our prospectively collected data provides robust preliminary findings which will be useful for others working in this relatively unexplored area of investigation.

In conclusion, this study reveals a high incidence of readmission and death among mother-newborn dyads following discharge from healthcare facilities. These occur within a broader context of very low rates of post-natal follow up and a high proportion of caesarean deliveries. Most complications were related to both maternal and neonatal infection suggesting that these may be detected and treated earlier on with improved discharge checks and follow-up care.

## Summary of recommendations

1. New patient-centered approaches to postnatal care are needed. Ideally this should be based on the individual risk of the mother-newborn dyad to manage unnecessary strain on health systems and out-of-pocket costs to families who may already be overburdened.
2. Postpartum women having undergone caesarean delivery are especially vulnerable and need individualized follow-up care.
3. Timing of postnatal care must be evidence based, and centered on when outcomes typically occur, to detect and treat complications early and reduce incidence of severe illness.
4. Postnatal care must be achievable, within the confines of the local context. Further research on the structural barriers to achieving postnatal care are urgently needed.

## Supporting information

S1 Table Demographics of dyads that were not discharged together (146 mothers, 134 neonates) or who were lost to follow-up (161 mothers, 162 neonates). (DOCX)

S2 Table Odds ratios for seeking maternal post-natal care and neonatal post-natal care from univariable and multivariable models for variables in the social and demographic factors domain. (DOCX)

S3 Table Odds ratios for seeking maternal post-natal care and neonatal post-natal care from univariable and multivariable models for variables in the pregnancy history and antenatal care domain. (DOCX)

S4 Table Odds ratios for seeking maternal post-natal care and neonatal post-natal care from univariable and multivariable models for variables in the admission and delivery domain. Birthweight was analysed as both a continuous and categorical variable with the continuous version being used in the multivariable model in the odds ratios presented for the other variables.
(DOCX)

S5 Table Odds ratios for seeking maternal post-natal care and neonatal post-natal care from univariable and multivariable models for variables in the maternal discharge domain. Delivery mode (from the delivery domain) was included as an additional covariate but is not reported in this table. MUAC was analysed as both a continuous and categorical variable with the continuous version being used in the multivariable model in the odds ratios presented for the other variables.
(DOCX)

S6 Table Odds ratios for seeking maternal post-natal care and neonatal post-natal care from univariable and multivariable models for variables in the neonatal discharge domain. Delivery mode was included as an additional covariate but is not reported in this table. Neonatal temperature and SpO2 were analysed as both a continuous and categorical variable with the continuous version being used in the multivariable model in the odds ratios presented for the other variables.
(DOCX)

S7 Table Characteristics of postpartum women who were readmitted. (DOCX)

S8 Table Characteristics of neonates who had either outcome (n = 133), re-admitted (n = 108), or died (n = 25). (DOCX)

S1 Checklist Inclusivity in global research. (DOCX)
