# Supplementary material for: The epidemiology and risk factors for postnatal complications among postpartum women and newborns in southwestern Uganda: A prospective cohort study
Source: PLOS Glob Public Health. 2024 Aug 7;4(8):e0003458. doi: 10.1371/journal.pgph.0003458 (PMC11305527; doi:10.1371/journal.pgph.0003458)
Supplement: S1 Table — (DOCX) [file pgph.0003458.s001.docx]

**Title: The epidemiology and risk factors for postnatal complications among postpartum women and neonates in Southwestern Uganda: a prospective cohort study**

**Supplementary Materials**

**Table S1.** Demographics of dyads that were not discharged together (146 mothers, 134 neonates) or who were lost to follow-up (161 mothers, 162 neonates).

| **Characteristics** | **Not discharged** | **Lost to follow-up** |
| --- | --- | --- |
| **Maternal Demographics** |  |  |
| Maternal age (years), mean (SD) | 25.6 (5.8) | 24.4 (5.6) |
| Parity, median (Q1, Q3) | 2 (1, 3) | 2 (1, 3) |
| Married, n (%) | 139 (95.2%) | 150 (95.5%) |
| Number of people living in household (excluding mother), median (Q1, Q3) | 2.5 (1, 4) | 2 (1, 3) |
| Education level, n (%) |  |  |
| *No school* | 12 (8.2%) | 12 (7.6%) |
| *P4-P7* | 68 (46.6%) | 71 (45.2%) |
| *S1-S6* | 44 (30.1%) | 51 (32.5%) |
| *Post Secondary* | 22 (15.1%) | 21 (13.4%) |
| Occupation with income, n (%) | 58 (39.7%) | 64 (40.8%) |
|  |  |  |
| **Maternal Diagnoses During Pregnancy n (%)** |  |  |
| HIV-infected | 18 (12.3%) | 22 (14%) |
| Gestational diabetes | 1 (0.7%) | 1 (0.6%) |
| Pre-eclampsia and eclampsia | 8 (5.5%) | 2 (1.3%) |
| Gestational hypertension | 9 (6.2%) | 0 (0%) |
| Antepartum hemorrhage | 16 (11%) | 7 (4.5%) |
| PPROM | 12 (8.2%) | 6 (3.8%) |
|  |  |  |
| **Pregnancy and Delivery** |  |  |
| Sought antenatal care from a doctor/nurse/midwife, n (%) | 144 (98.6%) | 157 (100%) |
| Number of antenatal care visits, median (Q1, Q3) | 4 (3, 4.8) | 4 (3, 5) |
| *<4 visits, n (%)* | 58 (39.7%) | 51 (32.5%) |
| *4-7 visits, n (%)* | 87 (59.6%) | 102 (65%) |
| *≥8 visits, n (%)* | 1 (0.7%) | 4 (2.5%) |
| Mode of delivery, n (%) |  |  |
| *Vaginal* | 57 (39%) | 88 (56.1%) |
| *Assisted vaginal* | 3 (2.1%) | 1 (0.6%) |
| *Caesarean with labour* | 64 (43.8%) | 57 (36.3%) |
| *Caesarean without labour* | 22 (15.1%) | 11 (7%) |
| Pregnancy <37 weeks, n (%) | 40 (27.4%) | 8 (5.1%) |
| Number of babies delivered, n (%) |  |  |
| *Individual* | 135 (92.5%) | 156 (99.4%) |
| *Twin* | 11 (7.5%) | 1 (0.6%) |
| *Triplet* | 0 (0%) | 0 (0%) |
|  |  |  |
| **Neonate Demographics** |  |  |
| Male sex, n (%) | 80 (59.7%) | 81 (51.3%) |
| Birth weight (kg), median (Q1, Q3) | 2.6 (1.9, 3.2) | 3.2 (3, 3.5) |
| Length (cm), median (Q1, Q3) | - | 49.8 (48, 50.9) |
| Apgar at 1 minute, median (Q1, Q3) | 7 (6, 9) | 9 (8, 9) |
| Apgar at 5 minutes, median (Q1, Q3) | 9 (8, 10) | 10 (10, 10) |

Abbreviations: PPROM = pre-term premature rupture of membranes; Q1 = first quartile; Q3 = third quartile; SD = standard deviation
