# Supplementary material for: The epidemiology and risk factors for postnatal complications among postpartum women and newborns in southwestern Uganda: A prospective cohort study
Source: PLOS Glob Public Health. 2024 Aug 7;4(8):e0003458. doi: 10.1371/journal.pgph.0003458 (PMC11305527; doi:10.1371/journal.pgph.0003458)
Supplement: S2 Table — (DOCX) [file pgph.0003458.s002.docx]

**Title: The epidemiology and risk factors for postnatal complications among postpartum women and neonates in Southwestern Uganda: a prospective cohort study**

**Supplementary Materials**

**Statistical Methods:**

Postnatal care for mother and newborn were assessed independently. Drivers were assessed using univariable and multivariable logistic regression within five prespecified domains of interest within the care continuum. Multivariable models included all variables within each domain.

**Supplementary Table S2.** Odds ratios for seeking maternal post-natal care and neonatal post-natal care from univariable and multivariable models for variables in the social and demographic factors domain.

| **Term (Reference Group)** | **N (%), Mean (SD), or Median (Q1, Q3)** | **N Missing (%)** | **Sought Maternal Post-Natal Care** | | **Sought Neonatal Post-Natal Care** | |
| --- | --- | --- | --- | --- | --- | --- |
|  |  |  | **Univariable OR** | **Multivariable OR** | **Univariable OR** | **Multivariable OR** |
| Married | 2751 (93.9%) | 0 (0%) | 0.89 (0.65, 1.22) | 1.05 (0.7, 1.57) | 1.42 (0.84, 2.27) | 1.27 (0.63, 2.51) |
| Lives with father | 2572 (87.8%) | 0 (0%) | 0.84 (0.67, 1.06) | 0.8 (0.6, 1.07) | 1.17 (0.78, 1.71) | 1.01 (0.57, 1.68) |
| Number of children in household | 2 (1, 3) | 0 (0%) | 0.98 (0.93, 1.02) | 0.97 (0.92, 1.02) | 0.98 (0.9, 1.06) | 0.99 (0.91, 1.08) |
| Employs a domestic worker in the home | 303 (10.3%) | 0 (0%) | 0.99 (0.77, 1.26) | 1.03 (0.79, 1.33) | **1.72 (1.04, 3.06)** | 1.57 (0.93, 2.83) |
| Anyone smokes at home | 130 (4.4%) | 1 (0.03%) | **1.48 (1.04, 2.11)** | **1.46 (1.02, 2.1)** | **0.55 (0.33, 0.97)** | 0.59 (0.35, 1.04) |
| Used herbs during pregnancy | 2249 (76.8%) | 0 (0%) | 1.06 (0.89, 1.27) | 0.88 (0.73, 1.07) | **0.65 (0.45, 0.92)** | **0.68 (0.46, 0.98)** |
| Sought care from traditional birth attendant | 866 (29.6%) | 0 (0%) | **1.45 (1.23, 1.7)** | **1.49 (1.25, 1.77)** | 1.03 (0.77, 1.39) | 1.2 (0.88, 1.65) |
| Education level ^a^ | 3 (2, 3) | 1 (0.03%) | 0.97 (0.89, 1.05) | 1.04 (0.94, 1.14) | **1.19 (1.02, 1.39)** | 1.07 (0.9, 1.28) |
| Paid occupation outside home | 1460 (49.8%) | 0 (0%) | 0.96 (0.83, 1.12) | 1.04 (0.88, 1.23) | 1.23 (0.94, 1.62) | 1.01 (0.75, 1.37) |
| Transport time ^b^ | 2 (1, 3) | 0 (0%) | **1.16 (1.08, 1.26)** | **1.15 (1.06, 1.25)** | **0.86 (0.75, 0.98)** | 0.94 (0.81, 1.09) |
| Transport mode (Motor transport) | 2630 (89.8%) | 0 (0%) |  |  |  |  |
| *Non-motor transport* | 76 (2.6%) |  | 0.62 (0.36, 1.03) | 0.63 (0.36, 1.04) | 1.2 (0.53, 3.46) | 1.08 (0.47, 3.12) |
| *Ambulance* | 224 (7.6%) |  | **1.59 (1.21, 2.1)** | **1.43 (1.07, 1.91)** | 0.82 (0.52, 1.35) | 1.07 (0.66, 1.8) |
| Someone other than mother decided to go hospital for delivery | 714 (24.4%) | 0 (0%) | 1.09 (0.91, 1.29) | 0.96 (0.8, 1.16) | **0.58 (0.44, 0.77)** | **0.63 (0.47, 0.86)** |

^a^ Analysed as continuous variable (1 = no school; 2 = P4-P7; 3 = S1-S6; 4 = Post secondary)

^b^ Analysed as continuous variable (1 = <30 minutes; 2 = 30-60 minutes; 3 = 1-2 hours; 4 = >2 hours)
