# Supplementary material for: The epidemiology and risk factors for postnatal complications among postpartum women and newborns in southwestern Uganda: A prospective cohort study
Source: PLOS Glob Public Health. 2024 Aug 7;4(8):e0003458. doi: 10.1371/journal.pgph.0003458 (PMC11305527; doi:10.1371/journal.pgph.0003458)
Supplement: S3 Table — (DOCX) [file pgph.0003458.s003.docx]

**Title: The epidemiology and risk factors for postnatal complications among postpartum women and neonates in Southwestern Uganda: a prospective cohort study**

**Supplementary Materials**

**Statistical Methods:**

Postnatal care for mother and newborn were assessed independently. Drivers were assessed using univariable and multivariable logistic regression within five prespecified domains of interest within the care continuum. Multivariable models included all variables within each domain.

**Supplementary Table S3.** Odds ratios for seeking maternal post-natal care and neonatal post-natal care from univariable and multivariable models for variables in the pregnancy history and antenatal care domain.

| **Term (Reference Group)** | **N (%), Mean (SD), or Median (Q1, Q3)** | **N Missing (%)** | **Sought Maternal Post-Natal Care** | | **Sought Neonatal Post-Natal Care** | |
| --- | --- | --- | --- | --- | --- | --- |
|  |  |  | **Univariable OR** | **Multivariable OR** | **Univariable OR** | **Multivariable OR** |
| Due date known | 2508 (85.6%) | 0 (0%) | **0.81 (0.66, 1)** | **0.77 (0.62, 0.95)** | 0.95 (0.64, 1.39) | 0.89 (0.59, 1.31) |
| Maternal age (18-35 years) | 2657 (90.7%) | 4 (0.14%) |  |  |  |  |
| *<18 years* | 79 (2.7%) |  | 0.68 (0.41, 1.09) | 0.73 (0.44, 1.2) | 0.85 (0.41, 2.05) | 0.88 (0.42, 2.15) |
| *>35 years* | 190 (6.5%) |  | 0.76 (0.55, 1.03) | **0.64 (0.45, 0.91)** | 0.63 (0.4, 1.03) | 0.64 (0.37, 1.14) |
| Parity | 2 (1, 3) | 0 (0%) | 1.03 (0.98, 1.07) | 1.05 (1, 1.11) | 0.96 (0.89, 1.04) | 1 (0.91, 1.09) |
| Prenatal vitamins during pregnancy | 2826 (96.5%) | 0 (0%) | 1.45 (0.95, 2.27) | 1.32 (0.86, 2.07) | 0.98 (0.43, 1.92) | 0.91 (0.4, 1.81) |
| History of chronic health conditions before pregnancy ^a^ | 2529 (86.3%) | 0 (0%) | 1.2 (0.96, 1.48) | 1.2 (0.87, 1.64) | 1.02 (0.7, 1.54) | 0.99 (0.58, 1.79) |
| HIV-infected | 309 (10.5%) | 0 (0%) | 1.17 (0.92, 1.49) | 0.94 (0.65, 1.35) | 1.02 (0.67, 1.62) | 1.13 (0.59, 2.2) |
| Malaria diagnosis during pregnancy | 331 (11.3%) | 0 (0%) | **1.4 (1.11, 1.77)** | 1.21 (0.92, 1.59) | 0.88 (0.59, 1.34) | 1.19 (0.73, 1.99) |
| Other pre-pregnancy complications ^b^ | 251 (8.6%) | 0 (0%) | 1.17 (0.89, 1.52) | 0.98 (0.72, 1.31) | 1.38 (0.83, 2.46) | 1.73 (0.98, 3.3) |
| Urinary tract infection during pregnancy | 892 (30.4%) | 0 (0%) | **1.33 (1.13, 1.56)** | 0.99 (0.77, 1.28) | 0.96 (0.72, 1.29) | 1.22 (0.76, 1.96) |
| Other pre-pregnancy infections | 256 (8.7%) | 0 (0%) | 1.04 (0.8, 1.35) | 0.89 (0.64, 1.22) | 1.08 (0.68, 1.81) | 1.42 (0.79, 2.66) |
| Previous admission | 262 (8.9%) | 1 (0.03%) | 1.09 (0.84, 1.41) | 0.87 (0.66, 1.16) | 0.69 (0.46, 1.07) | 0.65 (0.42, 1.05) |
| Diagnosed with condition during pregnancy ^c^ | 1441 (49.2%) | 0 (0%) | **1.41 (1.21, 1.64)** | **1.38 (1.03, 1.83)** | 0.9 (0.69, 1.18) | 0.68 (0.4, 1.16) |
| Number of antenatal care visits | 4 (4, 5) | 1 (0.03%) | **1.09 (1.04, 1.15)** | **1.09 (1.04, 1.15)** | **1.14 (1.04, 1.26)** | **1.15 (1.05, 1.28)** |

^a^ Includes diabetes, chronic hypertension, other heart disease, kidney disease, HIV, sickle cell, hepatitis B/C, tuberculosis, chronic mental illness, or other chronic health conditions (free text option)

^b^ Includes gestational diabetes, pre-eclampsia, eclampsia, gestational hypertension, antepartum hemorrhage/vaginal bleeding, PPROM, or preterm labour.

^c^ Includes gestational diabetes, pre-eclampsia, eclampsia, gestational hypertension, antepartum hemorrhage/ vaginal bleeding, PPROM, preterm labour, malaria, HIV, urinary tract infection, tuberculosis, anemia, or other infections (free text option).
