# Supplementary material for: The epidemiology and risk factors for postnatal complications among postpartum women and newborns in southwestern Uganda: A prospective cohort study
Source: PLOS Glob Public Health. 2024 Aug 7;4(8):e0003458. doi: 10.1371/journal.pgph.0003458 (PMC11305527; doi:10.1371/journal.pgph.0003458)
Supplement: S4 Table — Birthweight was analysed as both a continuous and categorical variable with the continuous version being used in the multivariable model in the odds ratios presented for the other variables. (DOCX) [file pgph.0003458.s004.docx]

**Title: The epidemiology and risk factors for postnatal complications among postpartum women and neonates in Southwestern Uganda: a prospective cohort study**

**Supplementary Materials**

**Statistical Methods:**

Postnatal care for mother and newborn were assessed independently. Drivers were assessed using univariable and multivariable logistic regression within five prespecified domains of interest within the care continuum. Multivariable models included all variables within each domain.

**Supplementary Table S4.** Odds ratios for seeking maternal post-natal care and neonatal post-natal care from univariable and multivariable models for variables in the admission and delivery domain. Birthweight was analysed as both a continuous and categorical variable with the continuous version being used in the multivariable model in the odds ratios presented for the other variables.

| **Term (Reference Group)** | **N (%), Mean (SD), or Median (Q1, Q3)** | **N Missing (%)** | **Sought Maternal Post-Natal Care** | | **Sought Neonatal Post-Natal Care** | |
| --- | --- | --- | --- | --- | --- | --- |
|  |  |  | **Univariable OR** | **Multivariable OR** | **Univariable OR** | **Multivariable OR** |
| Antibiotics during admission | 124 (4.2%) | 5 (0.17%) | **1.96 (1.37, 2.81)** | 1.1 (0.73, 1.68) | 1.12 (0.59, 2.4) | 1 (0.52, 2.2) |
| Episiotomy | 517 (17.6%) | 4 (0.14%) | **0.27 (0.21, 0.34)** | 0.92 (0.69, 1.23) | **0.72 (0.52, 1)** | 0.88 (0.62, 1.27) |
| Degree of tearing (None) | 2465 (84.1%) | 30 (1.02%) |  |  |  |  |
| *1* | 280 (9.6%) |  | **0.33 (0.24, 0.45)** | 1.18 (0.83, 1.65) | 0.78 (0.52, 1.21) | 0.95 (0.61, 1.52) |
| *2 or more* | 155 (5.3%) |  | **0.31 (0.2, 0.46)** | 1.09 (0.69, 1.66) | 0.75 (0.45, 1.32) | 0.95 (0.56, 1.71) |
| Labour induced | 108 (3.7%) | 1 (0.03%) | 0.73 (0.48, 1.1) | 1.07 (0.65, 1.72) | 0.83 (0.45, 1.73) | 0.99 (0.53, 2.08) |
| Labour obstructed | 280 (9.6%) | 108 (3.69%) | **3.77 (2.96, 4.81)** | 1.19 (0.89, 1.58) | 1.08 (0.71, 1.72) | 0.73 (0.45, 1.22) |
| Meconium in amniotic fluid | 519 (17.7%) | 142 (4.85%) | **1.68 (1.39, 2.02)** | 1.19 (0.95, 1.49) | 1.41 (0.98, 2.09) | 1.38 (0.95, 2.08) |
| Number of vaginal exams | 2 (1, 3) | 3 (0.1%) | 0.97 (0.93, 1.02) | 1 (0.95, 1.05) | **0.93 (0.87, 1)** | 0.94 (0.87, 1.01) |
| Caesarean delivery | 1168 (39.9%) | 0 (0%) | **10.8 (9.08, 12.89)** | **10.14 (8.01, 12.89)** | **1.67 (1.25, 2.25)** | **1.56 (1.01, 2.32)** |
| More than one baby delivered | 50 (1.7%) | 0 (0%) | 0.88 (0.56, 1.35) | 0.74 (0.43, 1.25) | 1.21 (0.57, 3.13) | 1.21 (0.54, 3.28) |
| Time from admission till delivery, per day | 0.3 (0.1, 0.7) | 6 (0.2%) | 1.03 (0.99, 1.1) | 1 (0.94, 1.07) | 0.96 (0.92, 1.03) | 0.95 (0.83, 1.03) |
| Time from admission till discharge, per day | 1.6 (1, 3.1) | 4 (0.14%) | **1.48 (1.4, 1.56)** | 1 (0.97, 1.04) | 1.02 (0.98, 1.1) | 1.02 (0.96, 1.15) |
| Time from labour till delivery, per day | 0.9 (0.5, 1.6) | 258 (8.81%) | **1.06 (1.02, 1.11)** | 0.98 (0.94, 1.03) | 1.04 (0.97, 1.17) | 1.03 (0.96, 1.17) |
| Baby resuscitated after birth | 496 (16.7%) | 373 (12.55%) | **1.28 (1.06, 1.55)** | 1.12 (0.89, 1.41) | 1.13 (0.8, 1.63) | 1.08 (0.75, 1.59) |
| Apgar score at 1 minute | 9 (8, 9) | 0 (0%) | **0.87 (0.8, 0.95)** | 1.04 (0.91, 1.19) | 0.95 (0.81, 1.1) | 0.97 (0.79, 1.2) |
| Apgar score at 5 minutes | 10 (10, 10) | 0 (0%) | **0.73 (0.64, 0.85)** | 0.81 (0.64, 1.01) | 0.91 (0.67, 1.18) | 1.02 (0.69, 1.47) |
| Visit was referred from another health centre | 1082 (36.9%) | 0 (0%) | **1.64 (1.41, 1.91)** | 1.19 (0.99, 1.43) | 1.2 (0.91, 1.6) | 1.11 (0.83, 1.5) |
| Delivery ≤37 weeks | 149 (5.1%) | 427 (14.57%) | 0.8 (0.58, 1.09) | 0.87 (0.59, 1.27) | 0.77 (0.48, 1.3) | 0.75 (0.46, 1.31) |
| Birthweight of baby, per kg | 3.2 (0.5) | 0 (0%) | **1.29 (1.1, 1.51)** | 0.94 (0.77, 1.14) | 0.93 (0.71, 1.23) | 0.87 (0.65, 1.18) |
| Birthweight of baby ≤2.5 kg | 232 (7.8%) | 0 (0%) | 0.85 (0.63, 1.12) | 1.14 (0.79, 1.63) | 1.07 (0.66, 1.85) | 1.15 (0.67, 2.1) |
