# Supplementary material for: The epidemiology and risk factors for postnatal complications among postpartum women and newborns in southwestern Uganda: A prospective cohort study
Source: PLOS Glob Public Health. 2024 Aug 7;4(8):e0003458. doi: 10.1371/journal.pgph.0003458 (PMC11305527; doi:10.1371/journal.pgph.0003458)
Supplement: S5 Table — Delivery mode (from the delivery domain) was included as an additional covariate but is not reported in this table. MUAC was analysed as both a continuous and categorical variable with the continuous version being used in the multivariable model in the odds ratios presented for the other variables. (DOCX) [file pgph.0003458.s005.docx]

**Title: The epidemiology and risk factors for postnatal complications among postpartum women and neonates in Southwestern Uganda: a prospective cohort study**

**Supplementary Materials**

**Statistical Methods:**

Postnatal care for mother and newborn were assessed independently. Drivers were assessed using univariable and multivariable logistic regression within five prespecified domains of interest within the care continuum. Multivariable models included all variables within each domain.

**Supplementary Table S5.** Odds ratios for seeking maternal post-natal care and neonatal post-natal care from univariable and multivariable models for variables in the maternal discharge domain. Delivery mode (from the delivery domain) was included as an additional covariate but is not reported in this table. MUAC was analysed as both a continuous and categorical variable with the continuous version being used in the multivariable model in the odds ratios presented for the other variables.

| **Term (Reference Group)** | **N (%), Mean (SD), or Median (Q1, Q3)** | **N Missing (%)** | **Sought Maternal Post-Natal Care** | | **Sought Neonatal Post-Natal Care** | |
| --- | --- | --- | --- | --- | --- | --- |
|  |  |  | **Univariable OR** | **Multivariable OR** | **Univariable OR** | **Multivariable OR** |
| Maternal systolic blood pressure | 112.4 (12.5) | 0 (0%) | **0.99 (0.98, 0.99)** | 0.99 (0.99, 1) | 1 (0.99, 1.01) | 1 (0.99, 1.02) |
| Maternal diastolic blood pressure | 70.6 (10) | 0 (0%) | **0.98 (0.97, 0.99)** | 1.01 (0.99, 1.02) | 0.99 (0.98, 1) | 0.99 (0.98, 1.01) |
| Maternal hypertension at discharge | 132 (4.5%) | 0 (0%) | 1 (0.69, 1.43) | 1.01 (0.61, 1.66) | 0.77 (0.45, 1.46) | 0.77 (0.38, 1.63) |
| Maternal temporal artery temperature, per degree | 36.5 (0.5) | 0 (0%) | **0.78 (0.66, 0.91)** | 0.96 (0.79, 1.16) | 0.79 (0.6, 1.04) | 0.8 (0.6, 1.07) |
| Maternal symptoms present ^a^ | 255 (8.7%) | 0 (0%) | **1.9 (1.47, 2.47)** | 1.14 (0.85, 1.55) | 1.22 (0.75, 2.11) | 1.1 (0.67, 1.91) |
| Able to start breastfeeding | 2907 (99.2%) | 0 (0%) | 1.1 (0.48, 2.75) | 0.83 (0.32, 2.35) | 1.11 (0.18, 3.82) | 1.06 (0.17, 3.67) |
| Maternal best SpO2 | 97.9 (1.3) | 0 (0%) | **0.92 (0.86, 0.97)** | 1 (0.93, 1.07) | 0.98 (0.88, 1.08) | 1 (0.89, 1.11) |
| Maternal best heart rate | 96.9 (16.4) | 0 (0%) | 1 (1, 1) | **1.01 (1, 1.01)** | 1 (0.99, 1.01) | 1.01 (1, 1.01) |
| Maternal respiratory rate | 20.8 (3.2) | 0 (0%) | 0.98 (0.96, 1) | 1 (0.97, 1.03) | **0.96 (0.92, 1)** | 0.96 (0.92, 1) |
| Maternal haemoglobin, per g/dL | 11.3 (2) | 1 (0.03%) | **0.88 (0.85, 0.92)** | 0.99 (0.95, 1.04) | 0.97 (0.91, 1.04) | 1 (0.93, 1.08) |
| Maternal random glucose | 5.8 (1.8) | 1 (0.03%) | 0.98 (0.94, 1.02) | 1 (0.95, 1.05) | 1.01 (0.94, 1.09) | 1.01 (0.93, 1.09) |
| MUAC, per mm | 279.9 (34.8) | 1 (0.03%) | 1 (1, 1) | 1 (1, 1) | 1 (0.99, 1) | 1 (0.99, 1) |
| *Maternal malnutrition, MUAC <230mm* | 112 (3.8%) | 1 (0.03%) | 1.23 (0.83, 1.81) | 1.12 (0.7, 1.77) | 0.69 (0.39, 1.34) | 0.68 (0.37, 1.34) |
| Time from delivery till discharge, per day | 1 (0.6, 2.6) | 9 (0.31%) | **1.92 (1.8, 2.06)** | 1 (0.96, 1.04) | **1.14 (1.03, 1.29)** | 1.02 (0.96, 1.16) |

^a^ Includes headache, visual changes, chest pain, shortness of breath, nausea with vomiting, abdominal pain on the right side, foul smelling vaginal discharge, stiff neck, or cough.
