# Supplementary material for: The epidemiology and risk factors for postnatal complications among postpartum women and newborns in southwestern Uganda: A prospective cohort study
Source: PLOS Glob Public Health. 2024 Aug 7;4(8):e0003458. doi: 10.1371/journal.pgph.0003458 (PMC11305527; doi:10.1371/journal.pgph.0003458)
Supplement: S6 Table — Delivery mode was included as an additional covariate but is not reported in this table. Neonatal temperature and SpO2 were analysed as both a continuous and categorical variable with the continuous version being used in the multivariable model in the odds ratios presented for the other variables. (DOCX) [file pgph.0003458.s006.docx]

**Title: The epidemiology and risk factors for postnatal complications among postpartum women and neonates in Southwestern Uganda: a prospective cohort study**

**Supplementary Materials**

**Statistical Methods:**

Postnatal care for mother and newborn were assessed independently. Drivers were assessed using univariable and multivariable logistic regression within five prespecified domains of interest within the care continuum. Multivariable models included all variables within each domain.

**Supplementary Table S6.** Odds ratios for seeking maternal post-natal care and neonatal post-natal care from univariable and multivariable models for variables in the neonatal discharge domain. Delivery mode (from the delivery domain) was included as an additional covariate but is not reported in this table. Neonatal temperature and SpO2 were analysed as both a continuous and categorical variable with the continuous version being used in the multivariable model in the odds ratios presented for the other variables.

| **Term (Reference)** | **N (%), Mean (SD), or Median (Q1, Q3)** | **N Missing (%)** | **Sought Maternal Post-Natal Care** | | **Sought Neonatal Post-Natal Care** | |
| --- | --- | --- | --- | --- | --- | --- |
|  |  |  | **Univariable OR** | **Multivariable OR** | **Univariable OR** | **Multivariable OR** |
| Male baby | 1524 (51.3%) | 3 (0.1%) | 1.05 (0.91, 1.22) | 1.01 (0.85, 1.21) | 1.09 (0.84, 1.43) | 1.09 (0.83, 1.44) |
| Baby defecated | 2668 (89.8%) | 3 (0.1%) | **1.85 (1.4, 2.46)** | 1.08 (0.74, 1.59) | 0.78 (0.46, 1.25) | 0.56 (0.3, 0.99) |
| Baby made urine | 2575 (86.6%) | 4 (0.13%) | **1.76 (1.37, 2.27)** | 1.27 (0.89, 1.8) | 1.24 (0.82, 1.8) | 1.53 (0.94, 2.41) |
| Neonatal length, per cm | 49.3 (2.4) | 9 (0.3%) | 0.99 (0.96, 1.02) | 0.96 (0.92, 1) | **0.94 (0.89, 0.99)** | **0.92 (0.86, 0.99)** |
| Neonatal head circumference, per cm | 35.1 (1.4) | 8 (0.27%) | **1.09 (1.03, 1.15)** | 1.02 (0.94, 1.1) | 1.01 (0.92, 1.11) | 1.03 (0.91, 1.17) |
| Neonatal weight, per kg | 3.1 (0.5) | 10 (0.34%) | 1.06 (0.9, 1.24) | 0.95 (0.7, 1.27) | 0.99 (0.74, 1.33) | 1.01 (0.64, 1.6) |
| Neonatal MUAC, per mm | 102 (9.8) | 21 (0.71%) | 1 (1, 1.01) | 1.01 (0.99, 1.02) | 1 (0.98, 1.01) | 1 (0.98, 1.03) |
| Neonatal respiratory rate | 53.9 (11.5) | 7 (0.24%) | 1 (0.99, 1.01) | 1 (0.99, 1.01) | 0.99 (0.98, 1) | 1 (0.98, 1.01) |
| Neonatal blood glucose | 3.7 (0.9) | 9 (0.3%) | 1.08 (0.99, 1.17) | 1.02 (0.92, 1.12) | 1.03 (0.89, 1.2) | 1.04 (0.89, 1.22) |
| Neonatal complications present ^a^ | 62 (2.1%) | 0 (0%) | 1.33 (0.84, 2.08) | 1.16 (0.68, 1.99) | 1.25 (0.55, 3.59) | 1.11 (0.49, 3.21) |
| Neonatal temporal artery temperature, per degree | 36.9 (0.6) | 7 (0.24%) | **1.24 (1.1, 1.39)** | 0.97 (0.84, 1.13) | 0.97 (0.79, 1.2) | 1.01 (0.81, 1.28) |
| *Normal, 36.5 to 37.5°C (reference)* | 1853 (62.3%) |  |  |  |  |  |
| *Hypothermic, <36.5°C* | 738 (24.8%) |  | **1 (0.84, 1.2)** | 1.18 (0.96, 1.46) | 1.08 (0.78, 1.5) | 1.05 (0.76, 1.47) |
| *Fever, >37.5°C* | 374 (12.6%) |  | **1.45 (1.16, 1.82)** | 1 (0.99, 1.01) | 0.99 (0.67, 1.52) | 1 (0.99, 1) |
| Weight change at discharge from birth, per kg | -0.1 (0.2) | 10 (0.34%) | **0.28 (0.19, 0.41)** | 1.19 (0.75, 1.88) | 1.34 (0.68, 2.6) | 1.85 (0.89, 3.75) |
| SpO_2_, % | 95.5 (94, 97) | 7 (0.24%) | **1.04 (1.02, 1.07)** | 1 (0.97, 1.03) | 1.04 (1, 1.08) | 1.04 (0.99, 1.08) |
| Hypoxia, SpO_2_ < 95% | 1056 (35.5%) | 7 (0.24%) | **0.77 (0.66, 0.9)** | 1.07 (0.81, 1.42) | 0.87 (0.66, 1.14) | 1.03 (0.67, 1.61) |
| Neonatal best heart rate | 135.4 (16.2) | 7 (0.24%) | 1 (1, 1.01) | 0.93 (0.77, 1.12) | 1 (0.99, 1) | 0.91 (0.69, 1.2) |

^a^ Includes increased or decreased tone, restlessness, irritability, or lethargy at assessment, bulging fontanelle, jaundice, abdominal distension.
