# Supplementary material for: The epidemiology and risk factors for postnatal complications among postpartum women and newborns in southwestern Uganda: A prospective cohort study
Source: PLOS Glob Public Health. 2024 Aug 7;4(8):e0003458. doi: 10.1371/journal.pgph.0003458 (PMC11305527; doi:10.1371/journal.pgph.0003458)
Supplement: S7 Table — (DOCX) [file pgph.0003458.s007.docx]

**Title: The epidemiology and risk factors for postnatal complications among postpartum women and neonates in Southwestern Uganda: a prospective cohort study**

**Supplementary Materials**

**Supplementary Table S7.** Characteristics of postpartum women who were readmitted

| **Characteristics** | **Re-admitted postpartum women (n=80)** |
| --- | --- |
| **Admission Symptoms, n (%)** |  |
| Abdominal pain | 45 (56.2%) |
| Abdominal tenderness when touches | 32 (40%) |
| Abnormal tiredness | 12 (15%) |
| Changes in vision | 5 (6.2%) |
| Convulsions | 2 (2.5%) |
| Cough (<14 days) | 1 (1.2%) |
| Cough (>14 days) | 1 (1.2%) |
| Diarrhea (<14 days) | 2 (2.5%) |
| Diarrhea (>14 days) | 1 (1.2%) |
| Fever (<7 days) | 34 (42.5%) |
| Fever (>7 days) | 2 (2.5%) |
| Foul smelling vaginal discharge | 23 (28.7%) |
| Heavy vaginal bleeding | 7 (8.8%) |
| Severe headache (<24 hours) | 23 (28.7%) |
| Shortness of breath | 3 (3.8%) |
| Vomiting | 4 (5%) |
| Other | 6 (7.5%) |
