# Supplementary material for: The epidemiology and risk factors for postnatal complications among postpartum women and newborns in southwestern Uganda: A prospective cohort study
Source: PLOS Glob Public Health. 2024 Aug 7;4(8):e0003458. doi: 10.1371/journal.pgph.0003458 (PMC11305527; doi:10.1371/journal.pgph.0003458)
Supplement: S8 Table — (DOCX) [file pgph.0003458.s008.docx]

**Title: The epidemiology and risk factors for postnatal complications among postpartum women and neonates in Southwestern Uganda: a prospective cohort study**

**Supplementary Materials**

**Supplementary Table S8.** Characteristics of neonates who had either outcome (n = 133), re-admitted (n = 108), or died (n = 25)

|  |  |  |  |
| --- | --- | --- | --- |
| **Admission Symptoms, n (%)** | **Either Outcome**  **(n=133)** | **Re-admitted**  **(n=108)** | **Died**  **(n=25)** |
| Abnormal sleeping | 5 (3.8%) | 5 (4.6%) | 0 (0%) |
| Blood in stool | 1 (0.8%) | 1 (0.9%) | 0 (0%) |
| Changes in urine color | 4 (3%) | 4 (3.7%) | 0 (0%) |
| Cough (<14 days) | 9 (6.8%) | 7 (6.5%) | 2 (8%) |
| Cough (>14 days) | 1 (0.8%) | 1 (0.9%) | 0 (0%) |
| Diarrhea (<14 days) | 3 (2.3%) | 3 (2.8%) | 0 (0%) |
| Diarrhea (>14 days) | 1 (0.8%) | 1 (0.9%) | 0 (0%) |
| Fever (<7 days) | 81 (60.9%) | 72 (66.7%) | 9 (36%) |
| Fever (>7 days) | 5 (3.8%) | 5 (4.6%) | 0 (0%) |
| Less urine than usual | 2 (1.5%) | 1 (0.9%) | 1 (4%) |
| Rash | 12 (9%) | 12 (11.1%) | 0 (0%) |
| Seizure/convulsions | 6 (4.5%) | 4 (3.7%) | 2 (8%) |
| Vomiting | 10 (7.5%) | 10 (9.3%) | 0 (0%) |
| Yellow soles | 10 (7.5%) | 10 (9.3%) | 0 (0%) |
| None of these symptoms | 12 (9%) | 10 (9.3%) | 2 (8%) |
| Unknown | 1 (0.8%) | 1 (0.9%) | 0 (0%) |
|  |  |  |  |
